# Monomeric *Camelus dromedarius* GSTM1 at low pH is structurally more thermostable than its native dimeric form

**DOI:** 10.1371/journal.pone.0205274

**Published:** 2018-10-10

**Authors:** Ajamaluddin Malik, Javed M. Khan, Salman F. Alamery, Dalia Fouad, Nikolaos E. Labrou, Mohamed S. Daoud, Mohamed O. Abdelkader, Farid S. Ataya

**Affiliations:** 1 Department of Biochemistry, Protein Research Chair, College of Science, King Saud University, Riyadh, Saudi Arabia; 2 Department of Food Science and Nutrition, Faculty of Food and Agricultural Sciences, King Saud University, Riyadh, Saudi Arabia; 3 Department of Zoology, College of Science, King Saud University, Riyadh, Saudi Arabia; 4 Department of Zoology and Entomology, Faculty of Science, Helwan University, Ein Helwan, Cairo, Egypt; 5 Laboratory of Enzyme Technology, Department of Biotechnology, School of Food, Biotechnology and Development, Agricultural University of Athens, Athens, Greece; 6 King Fahd Unit Laboratory, Department of Clinical and Chemical Pathology, Kasr Al-Ainy University Hospital, Cairo University, El-Manial, Cairo, Egypt; 7 Molecular Biology Department, Genetic Engineering Division, National Research Centre, Dokki, Giza, Egypt; Russian Academy of Medical Sciences, RUSSIAN FEDERATION

## Abstract

Glutathione S‒transferases (GSTs) are multifunctional enzymes that play an important role in detoxification, cellular signalling, and the stress response. *Camelus dromedarius* is well-adapted to survive in extreme desert climate and it has GSTs, for which limited information is available. This study investigated the structure-function and thermodynamic properties of a mu-class camel GST (*Cd*GSTM1) at different pH. Recombinant *Cd*GSTM1 (25.7 kDa) was expressed in *E*. *coli* and purified to homogeneity. Dimeric *Cd*GSTM1 dissociated into stable but inactive monomeric subunits at low pH. Conformational and thermodynamic changes during the thermal unfolding pathway of dimeric and monomeric *Cd*GSTM1 were characterised via a thermal shift assay and dynamic multimode spectroscopy (DMS). The thermal shift assay based on intrinsic tryptophan fluorescence revealed that *Cd*GSTM1 underwent a two-state unfolding pathway at pH 1.0–10.0. Its *Tm* value varied with varying pH. Another orthogonal technique based on far-UV CD also exhibited two-state unfolding in the dimeric and monomeric states. Generally, proteins tend to lose structural integrity and stability at low pH; however, monomeric *Cd*GSTM1 at pH 2.0 was thermally more stable and unfolded with lower van't Hoff enthalpy. The present findings provide essential information regarding the structural, functional, and thermodynamic properties of *Cd*GSTM1 at pH 1.0–10.0.

## Introduction

Glutathione S-transferase (GST; EC 2.5.1.18) is one of three major groups of enzymes that carry out detoxification (elimination of foreign, cytotoxic, and genotoxic compounds) and is found in virtually all organisms [1, 2]. GSTs belong to a supergene family of multifunctional enzymes and are grouped into different species-independent gene classes [3–5]. GSTs protect cellular macromolecules (proteins and nucleic acids) from various endogenous and exogenous electrophilic reactive compounds [6–8] and neutralize these toxic compounds by conjugating a thiol nucleophilic moiety from reduced glutathione (GSH, γ-glutamyl-cysteinyl-glycine) with electrophilic centres of the xenobiotic compounds [4, 7, 9]. Moreover, GSTs also regulate signalling pathways via protein-protein interactions [1, 10, 11]. GSTs are further sub-grouped into a cytosolic family and membrane-bound microsomal family [12–14]. Cytosolic GSTs are soluble and stable and consist of homo- or heterodimers with two-fold axes [3, 4, 15]. Cytosolic GST subunits are approximately 22–28 kDa in size and are divided into sixteen classes based on amino acid sequence similarity, substrate specificity, inhibitor sensitivity, and immunological cross-reactivity. Eight (Alpha [α], Mu [μ], Pi [π], Sigma [σ], Theta [θ], Omega [Ω], Kappa [κ], and Zeta [ζ]) of these 16 classes are found in mammals [12, 16–18].

GST class μ (GSTM; 51.5 kDa) is a homodimeric protein [19, 20]. Each subunit comprises two structural domains: the N-terminal domain (NTD) and the C-terminal domain (CTD). The NTD (1–81 residues) adopts a thioredoxin fold (βαβαββα motif), which is highly conserved through GST classes and contains a GSH-binding pocket. The CTD is larger (90–217 residues) and consists of 5 α-helices [3, 20–22]. The GSH-binding site is located on at the NTD; however, residues at the CTD interact with various hydrophobic xenobiotic substrates. Structural folds of the CTD are more variable than those of the NTD, which presumably facilitate specificity and binding of various structurally diverse xenobiotic compounds [3, 23].

Part of the active site is located on the dimer interface; therefore, GST dimerization is essential to for its activation [23, 24]. Moreover, interactions at the GST dimer interface also contribute to conformational stabilization, dynamics of the individual subunits, and cooperative behaviour between the two subunits of GSTs [25–32]. Hydrophobic NTD residues and charged CTD residues located at the dimer interface join the two subunits together [24, 26, 32, 33]. A cooperative two-state unfolding pathway has been reported in equilibrium unfolding studies on GST α1–1, π1–1, and Sj26GST [25, 34, 35], while a multistate pathway, with monomeric or even dimeric folding intermediates, has been reported in GST μ and σ [30, 36, 37].

*Camelus dromedarius* is adapted to extreme desert climate including elevated solar radiation, temperature, dryness, low nutrition, and scarcity of water [38, 39]. They are exposed to intrinsic and xenobiotic toxic agents that can damage cellular macromolecules (proteins and nucleic acids). Therefore, functional genomic characterisation of *C*. *dromedarius* genes involved in the stress response and adaptation is essential. Camel GSTM is an important detoxifying enzyme involved mediating survival and adaptation under stressful conditions.

In this study, recombinant camel GSTM1 was produced and purified from *E*. *coli*. Structural, functional, and thermodynamic characteristics and unfolding pathways of *Cd*GSTM1 were investigated at pH 1.0–10.0, using several advanced biophysical techniques.

## Results

### Multiple sequence alignment

**Fig 1:**
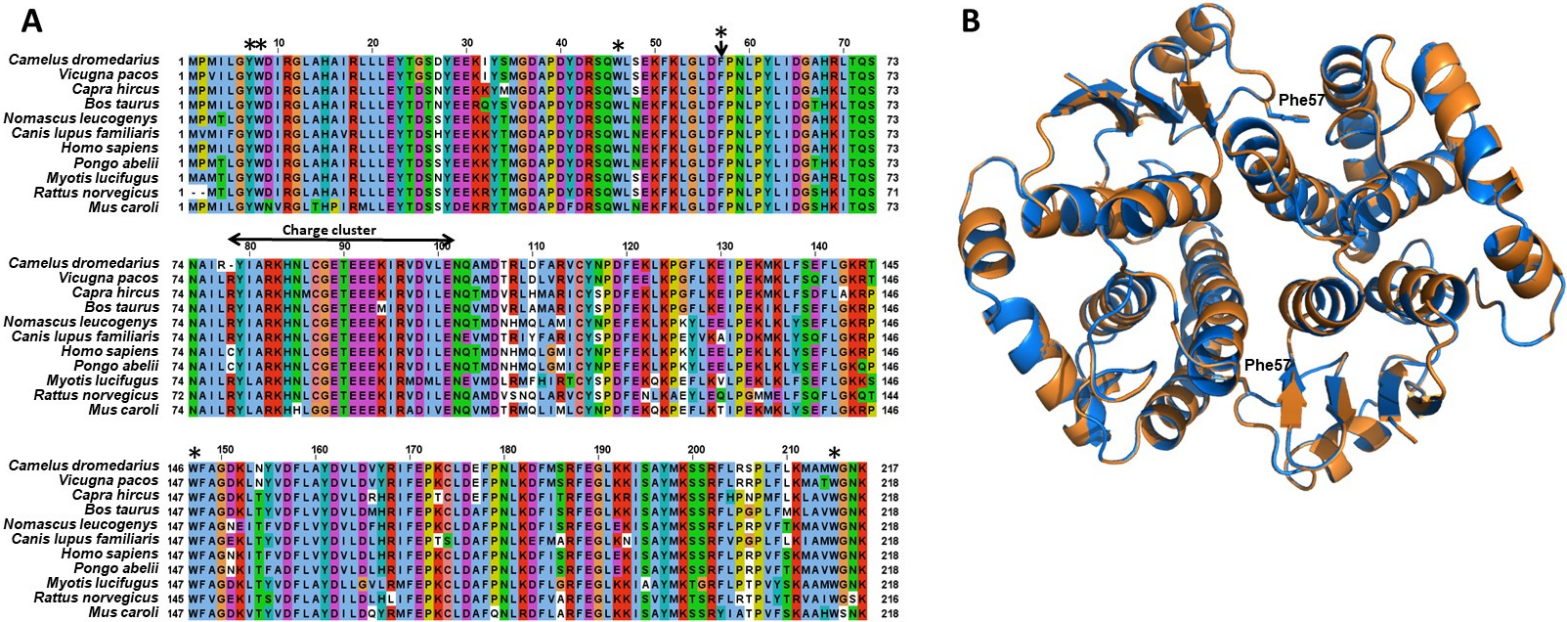
**(A) Multiple sequence alignment of homologous mu-class GSTs.** Alignment of *Cd*GSTM1 amino acid sequence with mammalian homologous mu-class GSTs. The alignment was generated by MAFFT Multiple Sequence Alignment algorithm. *C*. *dromedaries*, *V*. *pacos*, *C*. *hircus*, *B*. *taurus*, *N*. *leucogenys*, *C*. *l*. *familiaris*, *H*. *sapiens*, *P*. *abelii*, *M*. *lucifugus*, *R*. *norvegicus and M*. *caroli* were included in the alignment. Conserved Phe-57 at the interface indicated with an arrow. Mix charge cluster in a 24-residue-long stretch is labelled. The active-site residues involved in ligand interaction are labelled with an asterisk. (B) **Structural features of *Cd*GSTM1**. Superimposed 3D structure of *Cd*GSTM1 (orange) with *h*GSTM1 (blue). The superimposition indicated very high similarity between the conformation structures of *Cd*GSTM1 with *h*GSTM1. The hydrophobic lock-and-key forming residue (Phe57) is labelled.

The amino acid sequence of *Cd*GSTM1 was aligned with ten homologous mammalian GSTM1 by MAFFT Multiple Sequence Alignment [40] (Fig 1A). Multiple sequence alignment showed that *Cd*GSTM1 has the highest sequence identity with *V*. *pacos* GSTM1 (96%). *Cd*GSTM1 showed 96, 86, 85, 81, 80, 80, 80,78, 78 and 75% identity with *V*. *pacos* GSTM1, *C*. *hircus* GSTM1, *B*. *Taurus* GSTM1, *C*. *l*. *familiaris* GSTM1-1, *N*. *leucogenys* GSTM1-1, *H*. *sapiens* GSTM1-1, *M*. *lucifugus* GSTM1-3, *P*. *abelii* GSTM1-2, *R*. *norvegicus* GSTM3, *M*. *caroli* GSTM1, respectively (Fig 1A). The amino acid sequence alignment showed active site residues and dimer interface charge cluster are highly conserved (Fig 1A). Due to unavailability of X-ray/NMR structure of *Cd*GSTM1, the 3D structure of CdGSTM1 was modeled using 2.68 Å X-ray diffraction structure of ligand-free hGSTM1A-1A (PDBID: 1GTU) as a template. Modeled 3D structure of *Cd*GSTM1 was superimposed on hGSTM1A-1A (Fig 1B), indicated a high degree of similarity.

### Purification and determination of the quaternary structure of recombinant *Cd*GSTM1

**Fig 2:**
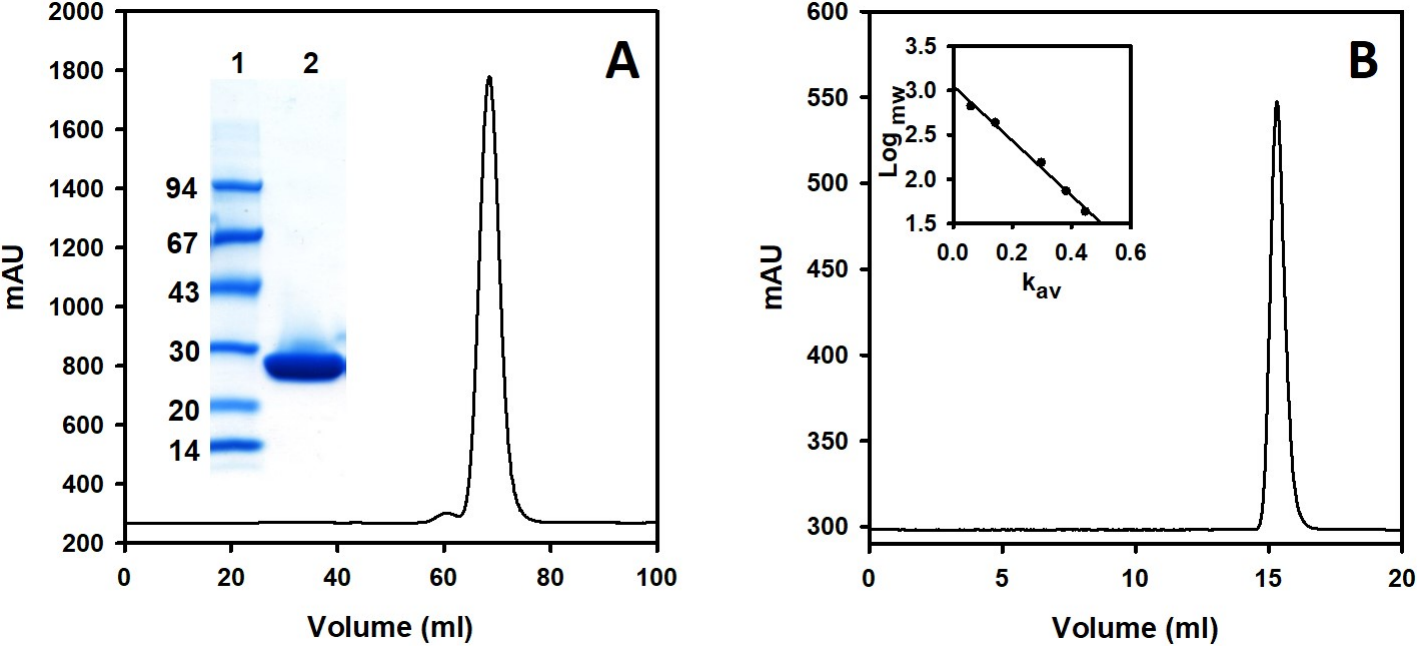
Purification and characterization of *Cd*GSTM1. (A) Pooled active fractions from reduced glutathione-agarose was loaded on a Superdex 75 column. *Cd*GSTM1 was eluted at a single symmetrical peak. Purity analysis on sodium dodecyl sulphate polyacrylamide gel electrophoresis is shown in the *inset*. Lane 1 contains a low-molecular-weight marker; lane 2, the pool of size-exclusion chromatography (SEC) fractions. (B) Molecular weight determination of *Cd*GSTM1 is shown. Superdex 200 increase column was calibrated with different molecular weight proteins, shown in the inset. SEC-purified protein was loaded onto a calibrated Superdex 200 increase column. The molecular weight of *Cd*GSTM1 was determined from its elution volume (Ve).

*Cd*GSTM1 was over-expressed and purified from *E*. *coli*. Active GSH-agarose elution fractions were pooled. The pooled fraction was further purified via size-exclusion chromatography to eliminate impurities and GSH (Fig 2A). A single sharp peak was observed at 70 mL of eluted volume which is indicated that the fractions contained a homogenous population of dimeric *Cd*GSTM1. Sodium dodecyl sulphate polyacrylamide gel electrophoresis (SDS-PAGE) was also performed to confirm the level of *Cd*GSTM1 homogeneity (purity). SDS-PAGE revealed a single band at ~26 kDa, thereby confirming that *Cd*GSTM1 was highly pure (Fig 2A inset). The yield of soluble, pure *Cd*GSTM1 protein was approximately 30 mg L^-1^ in a shake-flask culture. The quaternary structure of the purified protein was determined using an analytical Superdex 200 column. *Cd*GSTM1 was eluted at a volume of 15.3 mL, which corresponds to approximately 50.58 kDa which, is similar to the calculated molecular weight (51.5 kDa) of dimeric *Cd*GSTM1 (Fig 2B).

### Effect of pH change on *Cd*GSTM1 activity

**Fig 3:**
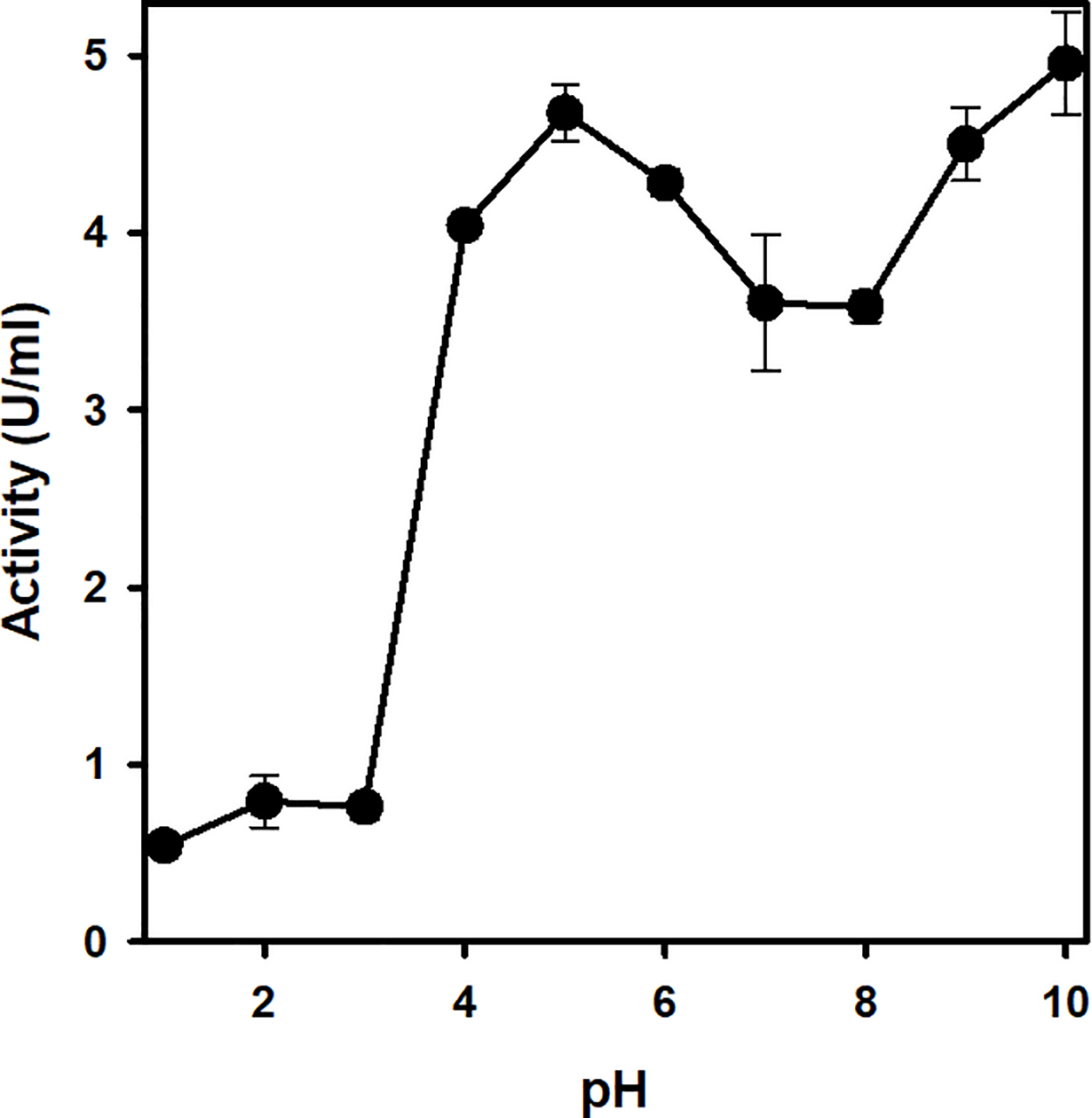
Effect of pH on *Cd*GSTM1 activity. *Cd*GSTM1 (0.2 mg mL^-1^) was incubated overnight in 50 mM buffers with various pH ranges (pH 1.0 to 10.0) with a pH 1.0 interval. Residual GST activity was measured at 25°C in 100 mM phosphate buffer (pH 6.5) containing 1 mM GSH and 1 mM 2,4-dinitrochlorobenzene.

The enzymatic activity of *Cd*GSTM1 was measured via equilibration at pH 1.0 to 10.0, and subsequently, residual activity was evaluated using 2,4-dinitrochlorobenzene (CDNB) as a substrate at optimum pH of 6.5 [41]. As shown in Fig 3, enzymatic activity of *Cd*GSTM1 drastically decreased below pH 4.0, probably owing to denaturation or dissociation of *Cd*GSTM1 subunits. However, loss of enzymatic activity at acidic pH was reversible (data not shown).

### Effect of pH change on *Cd*GSTM1 conformation

**Fig 4:**
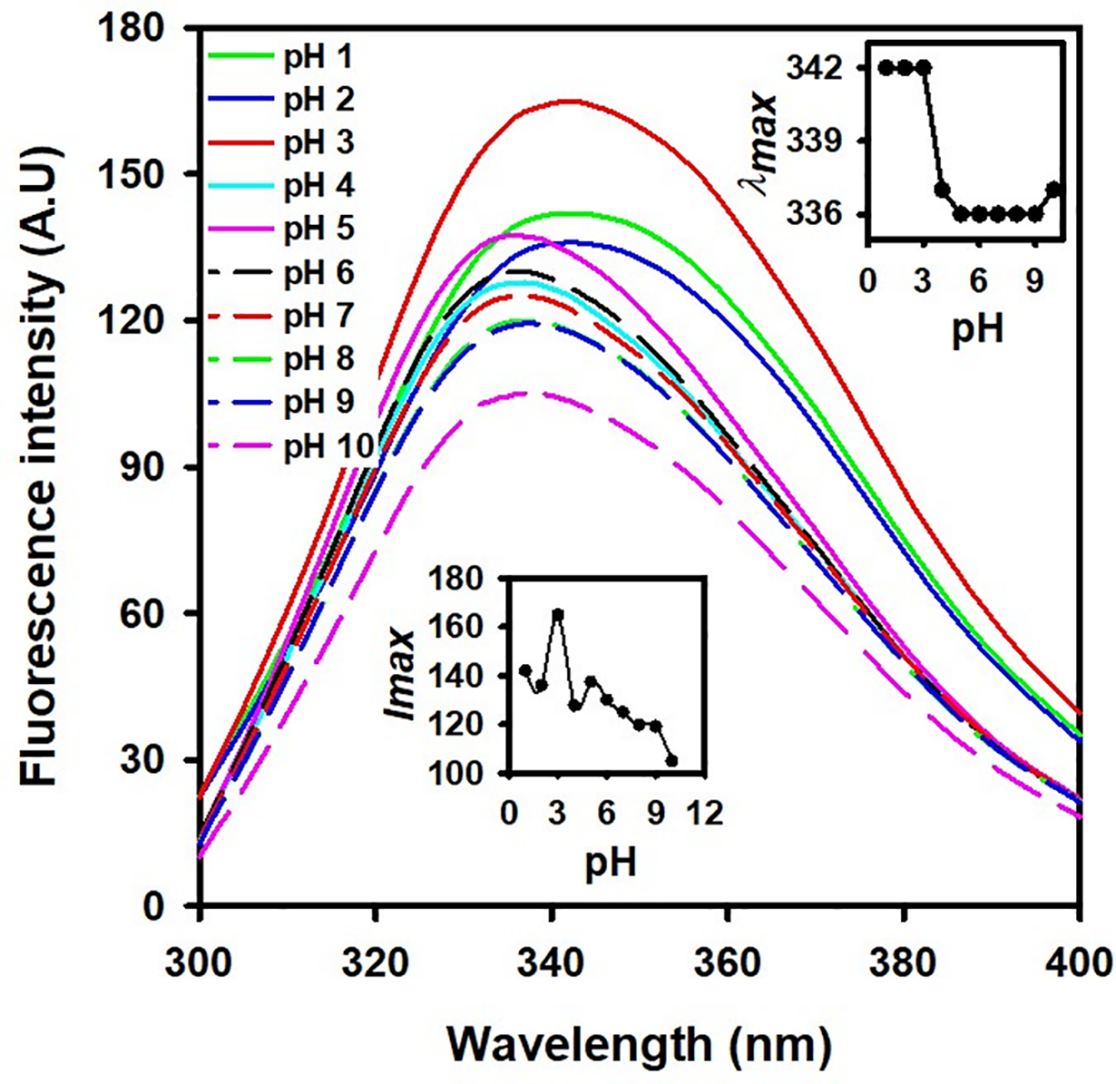
Total fluorescence spectra of *Cd*GSTM1 at different pH values. *Cd*GSTM1 (0.1 mg mL^-1^) was excited at 280 nm at different pH values. Data collected from 300 to 400 nm at 22°C are shown. Each spectrum at different pH is colour-coded. *λmax* and *Imax* are plotted with respect to pH, shown in the *insets*.

Far-UV CD, fluorescence spectroscopy, and SEC were performed to investigate changes in the secondary, tertiary, and quaternary structure of *Cd*GSTM1 at different pH values. Before the measurements, *Cd*GSTM1 was incubated overnight at different pH values ranging from 1.0–10.0. Intrinsic fluorescence measurements provided clear information regarding protein tertiary structural change. The primary intrinsic fluorophores in proteins are tryptophan (Trp), tyrosine, and phenylalanine. Total fluorescence spectra of *Cd*GSTM1 at different pHs are shown in Fig 4. From the figure, it was recorded that the *Cd*GSTM1 at pH 7.0 showed maximum fluorescence intensity at 336 nm, confirming that *Cd*GSTM1 exists in a well-folded form. The fluorescence emission maximum of *Cd*GSTM1 remained unchanged in the range of 5.0–9.0 pH, indicating that the tertiary structure of *Cd*GSTM1 was intact (Fig 4, inset). However, between pH 3.0 and 5.0, the wavelength maximum of *Cd*GSTM1 was 6.0 nm (red shifted). The red shift in the wavelength maximum signified that the tryptophan residues were exposed to the polar environment, thus indicating changes in protein tertiary structure. Moreover, the maximum wavelength remained unchanged below pH 3.0, indicating that the same microenvironment around tryptophan residues was retained. Fluorescence intensity of *Cd*GSTM1 increased gradually with a reduction in pH because the position of certain amino acid residues changed apart from the Trp residues, which were responsible for quenching of Trp fluorescence. Such pH-dependent changes were also reported previously [42]. The shift in maximum wavelength suggests that dimeric *Cd*GSTM1 either begins monomerising below pH 5.0 or undergoes a loss of tertiary structure interactions, which were further characterized by other advance techniques.

### Changes in the secondary structure of *Cd*GSTM1 with changes in pH

**Fig 5:**
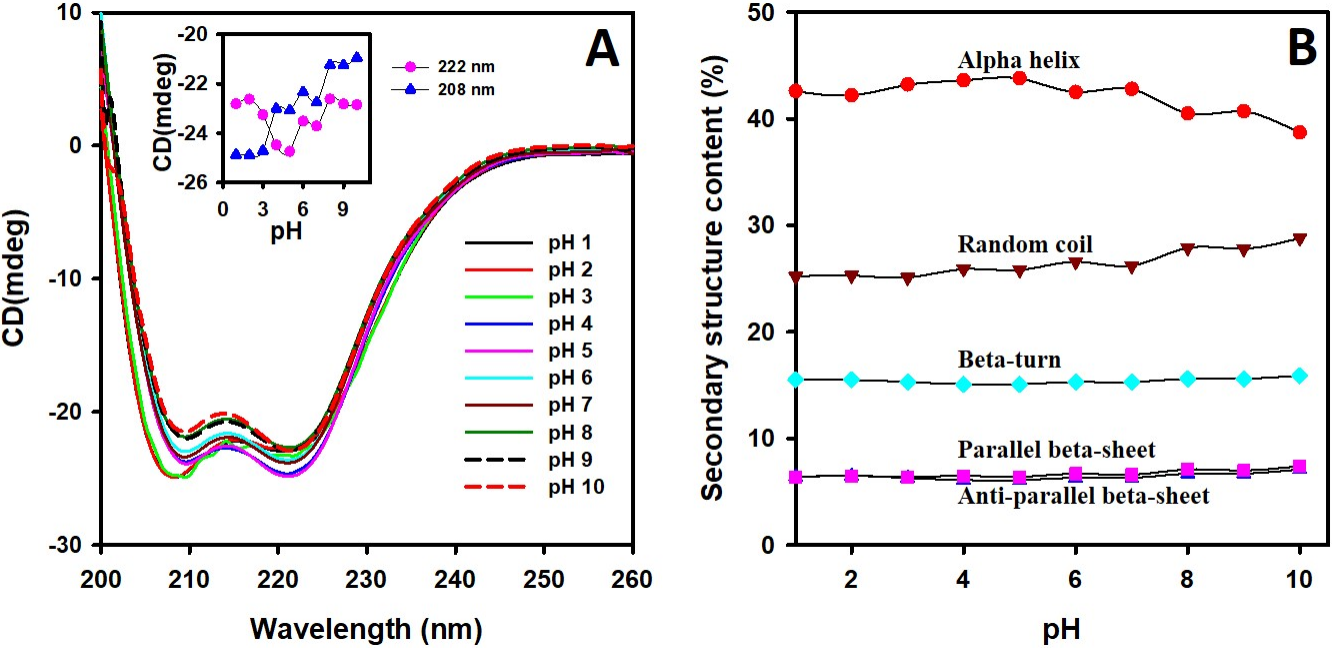
Far-UV circular dichroism (CD) spectra of *Cd*GSTM1 at different pH values. (A). Far-UV CD spectra of 0.2 mg mL^-1^ *Cd*GSTM1 at pH 1.0 to 10.0. Changes in ellipticities at 208 and 222 nm at different pH are plotted in the *inset* figure. (B) Relative changes in the *Cd*GSTM1 secondary structure contents (%) at different pH values were calculated via CDNN. Each structure labelled on the curve.

Far-UV CD was used to characterize the change in the secondary structure of *Cd*GSTM1 with respect to change in pH. The secondary structure of *Cd*GSTM1 at different pH values was analysed via far-UV CD (200–250 nm) measurements (Fig 5). Far-UV CD spectra of *Cd*GSTM1 at pH 7.0 yielded two negative minima, one at 208 and the other at 222 nm, which is a characteristic feature of an alpha helix (Fig 5A). Changes in the negative ellipticity of *Cd*GSTM1 at 208 and 222 nm were insignificant at pH 1.0–10.0. CDNN analysis revealed that percent secondary structure contents (alpha-helical, beta-sheet, beta-turn, and random coil) of *Cd*GSTM1 were not significantly altered with changes in pH (Fig 5B). Far-UV CD results indicated that the secondary structure of *Cd*GSTM1 was remained stable with changes in pH.

### The 8-anilino-1-naphthalenesulfonic acid (ANS) binding assay

**Fig 6:**
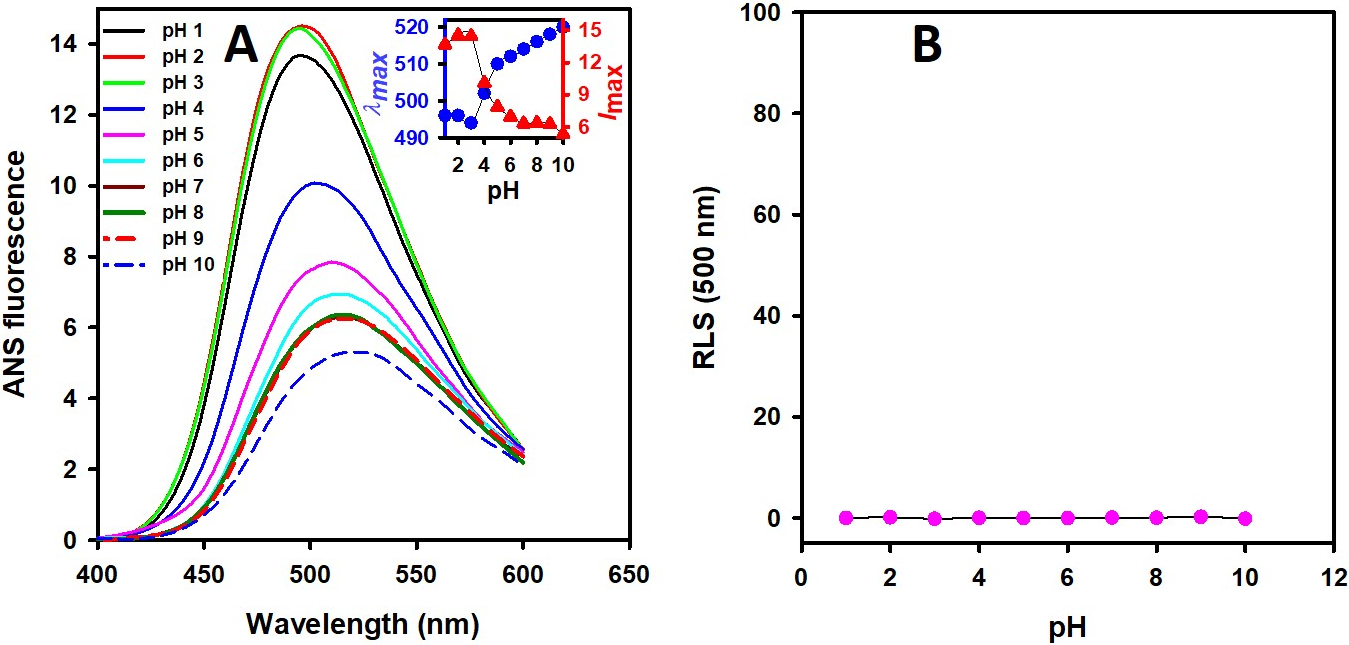
**(A) Effect of pH on the binding of 8-anilino-1-naphthalenesulfonic acid (ANS) with *Cd*GSTM1.** The binding of ANS (200 μM) with *Cd*GSTM1 (4 μM) at pH 1.0–10.0 was monitored by excitation at 380 nm (bandwidth 2.5 nm) and emission spectra collected from 400–600 nm (bandwidth 5 nm) at 22°C. **(B)** Rayleigh light scattering measurements of *Cd*GSTM1 (0.1 mg mL^-1^) were obtained at 500 nm wavelength at pH 1.0–10.0.

Exposure of hydrophobic patches in *Cd*GSTM1 at different pH were characterized by ANS fluorescence measurements. ANS is a hydrophobic, sensitive dye, which binds with hydrophobic regions of proteins. At pH 7.4, the *Cd*GSTM1 displayed maximum fluorescence intensity at 518 nm, which confirms that *Cd*GSTM1 is well-folded, while at an acidic pH (pH 2.0) the fluorescence intensity was increased by approximately two folds with maximum wavelength blue-shifted up to 10 nm (Fig 6A). Increase in ANS fluorescence intensity and blue-shift in maximum wavelength signified that the *Cd*GSTM1 was either partially unfolded or monomerised.

### Rayleigh light scattering measurements

Rayleigh light scattering was performed to investigate pH-dependent *Cd*GSTM1 aggregation. Light scattering of samples was monitored at 500 nm to determine the aggregation behaviour of *Cd*GSTM1 at different pH values. As shown in Fig 6B, light scattering was insignificant at all the pHs values. Thus, *Cd*GSTM1 does not form aggregates at any pH.

### Gel-permeation chromatography

**Fig 7:**
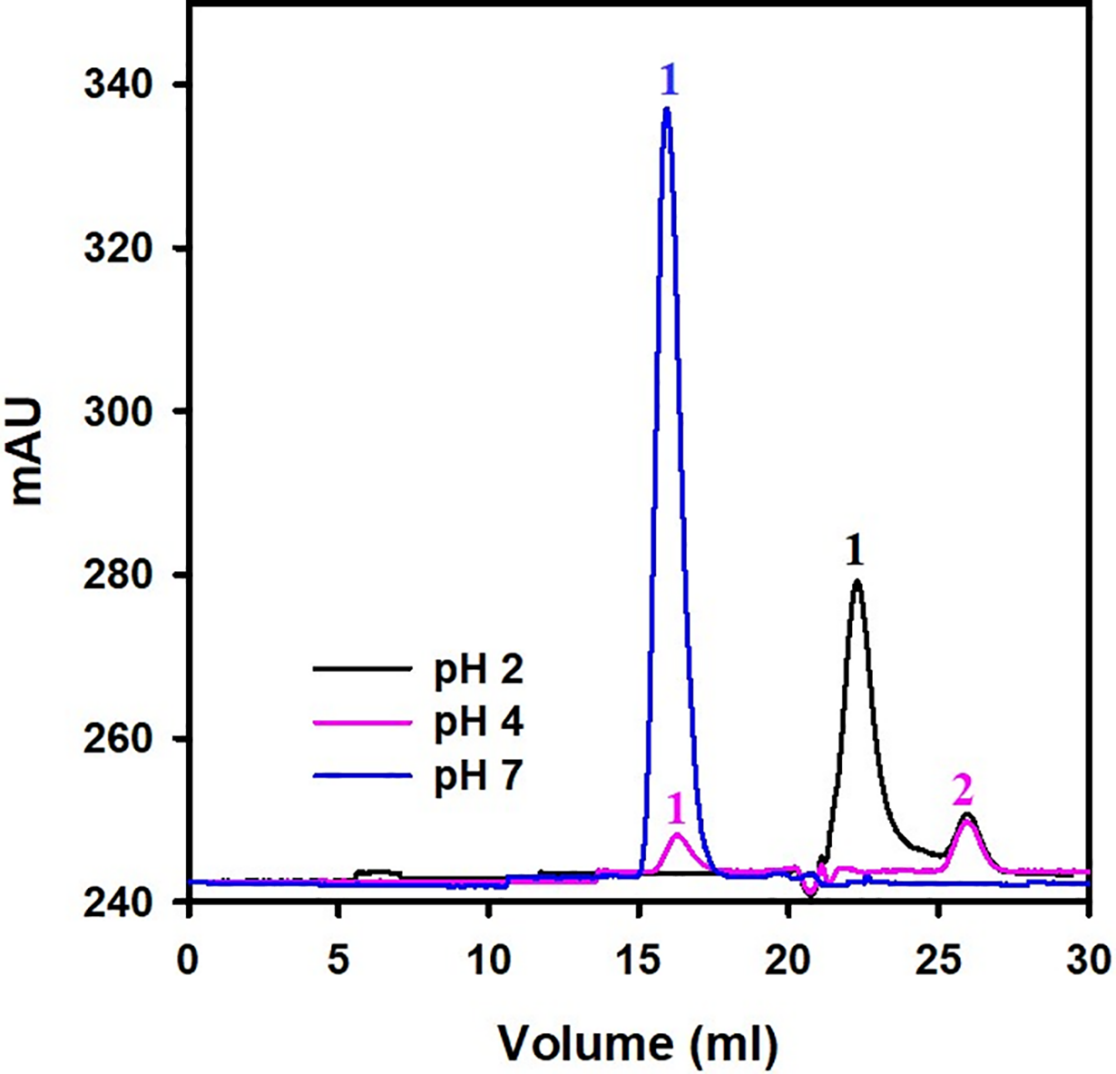
Determination of the quaternary structure of *Cd*GSTM1 at different pH values. *Cd*GSTM1 (0.25 mg) at pH 2.0, 4.0, and 7.0 was passed through calibrated Superdex 200 increase column. *Cd*GSTM1 at pH 7.0 eluted in a single peak corresponding to the dimeric form (blue) is shown. At slight acidic pH 4.0 (pink), a small peak corresponding to dimer and one peak of monomeric size was observed. At acidic pH 2.0 (black), monomeric protein in two populations was detected.

Gel-permeation chromatography is used to separate proteins on the basis of size and it is also used to determine the changes in the quaternary structure of proteins. Intrinsic and ANS fluorescence analyses revealed that camel *Cd*GSTM1 undergoes a sharp transition between pH 3.0 and 5.0, probably owing to pH-dependent monomerisation or partial unfolding. Therefore, gel-permeation chromatography was performed to distinguish the level of monomerisation at pH 2.0 (after transition), 4.0 (during the transition), and 7.0 (before the transition; Fig 7). At pH 7.0, *Cd*GSTM1 was eluted at a single symmetrical peak (blue), corresponding with its dimeric state (Fig 2B). When *Cd*GSTM1 was equilibrated at pH 4.0 (pink), gel-permeation chromatograms revealed two peaks, one at 16.3 mL and the other at 25.9 mL, indicating that two types of populations were obtained: one with the size of the dimeric state; the other, the monomeric state. *Cd*GSTM1 incubated at pH 2.0 yielded peaks at 22.2 and 25.9 mL similar to those of the monomeric state (black). Gel-permeation chromatography revealed that above pH 5.0, *Cd*GSTM1 exists in its dimeric form; however, between pH 3.0 and 5.0, it exists in a monomeric-dimeric equilibrium and below pH 3.0, it exists only in the monomeric form.

### Glutaraldehyde cross-linking

**Fig 8:**
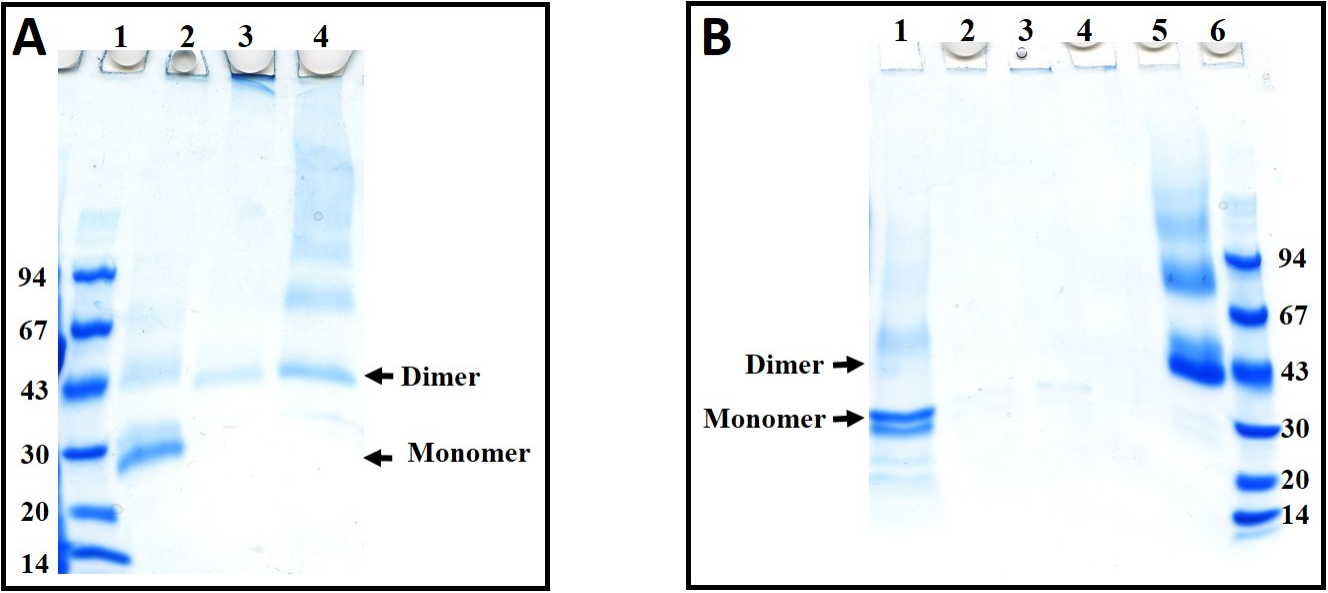
Sodium dodecyl sulphate polyacrylamide gel electrophoresis profiles of glutaraldehyde cross-linked *Cd*GSTM1at pH 2.0, 4.0, and 7.0 before and after size-exclusion chromatography (SEC). **(A)** Crosslinking before SEC. Lane 1, low-molecular-weight marker; lane 2, pH 2.0, cross-linked before SEC; lane 3, pH 4.0, cross-linked before SEC; lane 4, pH 7.0, cross-linked before SEC. **(B)** Crosslinking after SEC. Lane 1, pH 2.0, peak 1, cross-linked After SEC; lane 2, pH 2.0, peak 2, cross-linked After SEC; lane 3, pH 4.0, peak 1, cross-linked After SEC; lane 4, pH 4.0, peak 2, cross-linked After SEC; lane 5, pH 7.0, peak 1, cross-linked After SEC; lane 6, low-molecular-weight marker.

Glutaraldehyde cross-linking is widely used techniques to distinguish the subunit composition (monomer, dimer and tetramer) of proteins. pH-dependent changes in the quaternary structure of *Cd*GSTM1 were further investigated via glutaraldehyde cross-linking (Fig 8). *Cd*GSTM1 treated at pH 2.0, 4.0, and 7.0 was passed through a Superdex 200 column to separate *Cd*GSTM1 monomers from dimers (Fig 7). The samples of *Cd*GSTM1 at pH 2.0, 4.0, and 7.0 before and after gel filtration were cross-linked with glutaraldehyde. The cross-linked products of *Cd*GSTM1 before (Fig 8A) and after (Fig 8B) gel filtration were further analysed via SDS-PAGE. As shown in Fig 8A, *Cd*GSTM1 at pH 2.0 (lane 2) showed a predominant band at the monomeric size of *Cd*GSTM1 (dissociated state). *Cd*GSTM1 at pH 4.0 (lane 3) exists as a dimer and dissociated form, which cross-links with high-molecular-weight aggregates (which remained at the top of the gel). At pH 7.0 (lane 4), a prominent band corresponding to the dimeric form of *Cd*GSTM1 was observed.

*Cd*GSTM1 at different pH was loaded on a 24-mL bed volume Superdex 200 column to separate monomer-dimer species of *Cd*GSTM1. At pH 2.0, *Cd*GSTM1 was dissociated and eluted at two peaks (22.2 and 25.9 mL). Although 150 mM NaCl was added in the gel filtration buffer to suppress protein-matrix interactions, *Cd*GSTM1at pH 2.0 was retained in the column and eluted near the column bed volume. Similarly, *Cd*GSTM1 at pH 4.0, where it exits in a monomer-dimer equilibrium, eluted at two peaks. The dimer eluted at the expected peak (16.3 mL), followed by the monomeric form after crossing the bed volume owing to retention in the matrix. All elution peaks obtained at pH 2.0, 4.0, and 7.0 were separately subjected to glutaraldehyde cross-linkage (Fig 8B). The first peak at pH 2.0 displayed cross-linkage in the monomeric form (lane 1) and the second peak was very small and showed a faint band corresponding to the monomeric size of *Cd*GSTM1 (lane 2). At pH 4.0, *Cd*GSTM1 was eluted into very small peaks, which yielded very faint bands on SDS-PAGE (lane 3 and 4). *Cd*GSTM1 at pH 7.0 eluted at the dimeric size and the cross-linked form revealed the predominant dimeric state (lane 5). Furthermore, all peaks at different pH values were subjected to trichloroacetic acid precipitation and analysed via SDS-PAGE, thereby revealing the monomeric size of *Cd*GSTM1 (data not shown).

### Acrylamide quenching fluorescence

**Fig 9:**
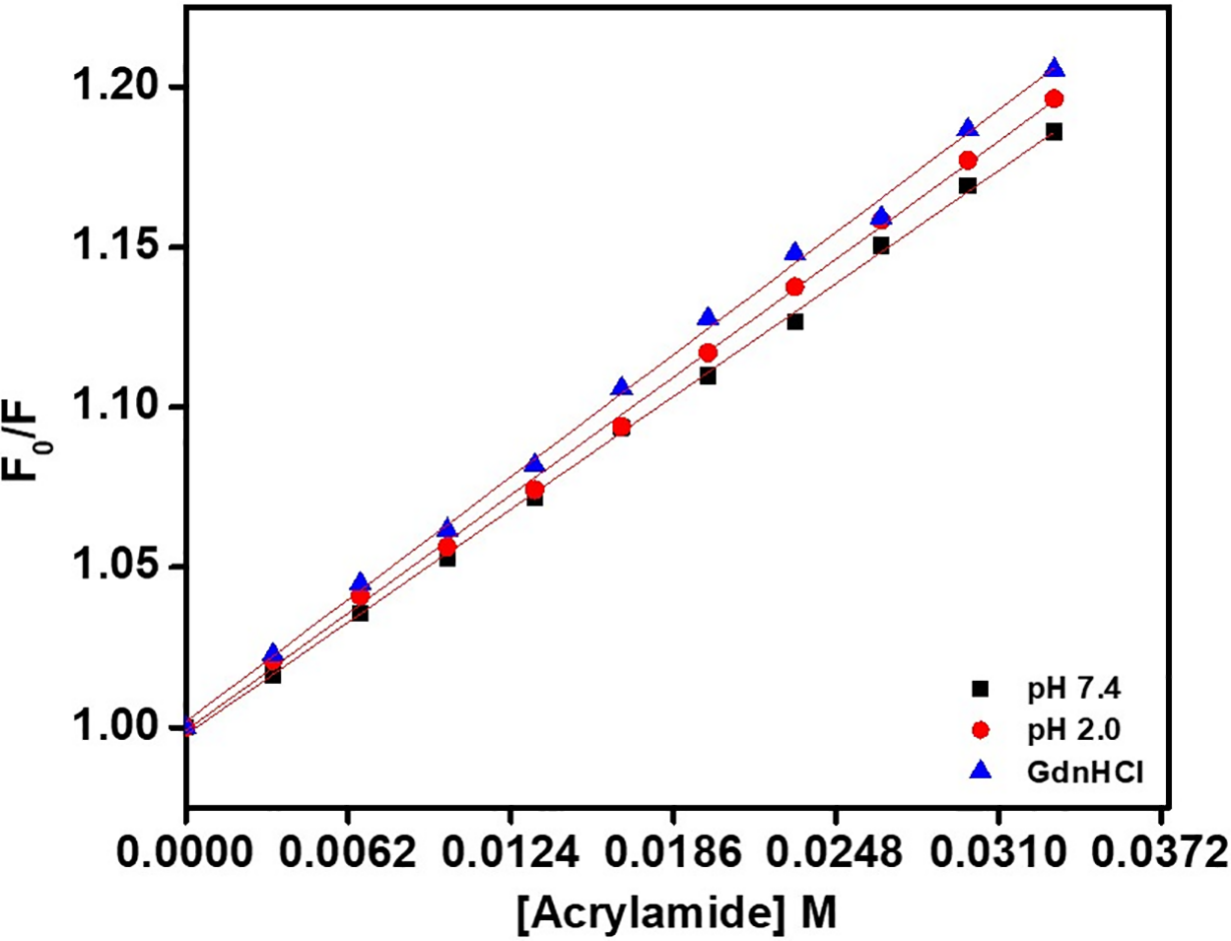
Identification of exposure of tryptophan residues via acrylamide quenching. The Stern-Volmer Plot from acrylamide quenching of *Cd*GSTM1 at native pH (7.4) (black square), acidic pH (2.0) (red circle) and in the presence of 6M GdnHCl (blue triangle).

Acrylamide quenching measurements were done to characterize the Trp exposure of proteins. The exposure of Trp amino acid residues to polar solvents was identified via uncharged acrylamide quenching measurements. The present results provide a detailed insight into conformational changes in *Cd*GSTM1 protein at different pH conditions. Fig 9 depicts the Stern-Volmer plot for *Cd*GSTM1 protein at different pH values. The Stern-Volmer quenching constant (Ksv) values are shown in Table 1. As shown in Fig 9 and Table 1, the Ksv values in the native state at an acidic pH and in the presence of 6M GdnHCl are as follows: 5.68 (pH 7.4), 5.93 (pH 2.0), and 6.16 M (6M GdnHCl). The Ksv values of *Cd*GSTM1 at pH 7.4 were markedly low because of complete folding of *Cd*GSTM1 and the Ksv values at 6M GdnHCl denaturation of *Cd*GSTM1 were the highest because of higher exposure of Trp residues owing to complete unfolding of the *Cd*GSTM1 protein. However, Trp residues were also exposed at pH 2.0, but to a lesser extent than in 6M GdnHCl-treated samples. Hence, *Cd*GSTM1 exists in a well-folded form at a native pH, slightly unfolded at low pH, and completely unfolded in the presence of 6M GdnHCl. The slight exposure of Trp residues at pH 2.0 suggests that either *Cd*GSTM1 is partially unfolded or the subunit dissociation.

**Table 1:** Acrylamide quenching constant (Ksv) values at three different conditions.

S.No.	Conditions	*K*_*sv*_M^-1^	R^2^
1	pH 7.4	5.68	0.997
2	pH 2.0	5.93	0.993
3	6M GdnHCl	6.16	0.999

### Thermal stability of *Cd*GSTM1 at different pH, determined via the thermal shift assay

**Fig 10:**
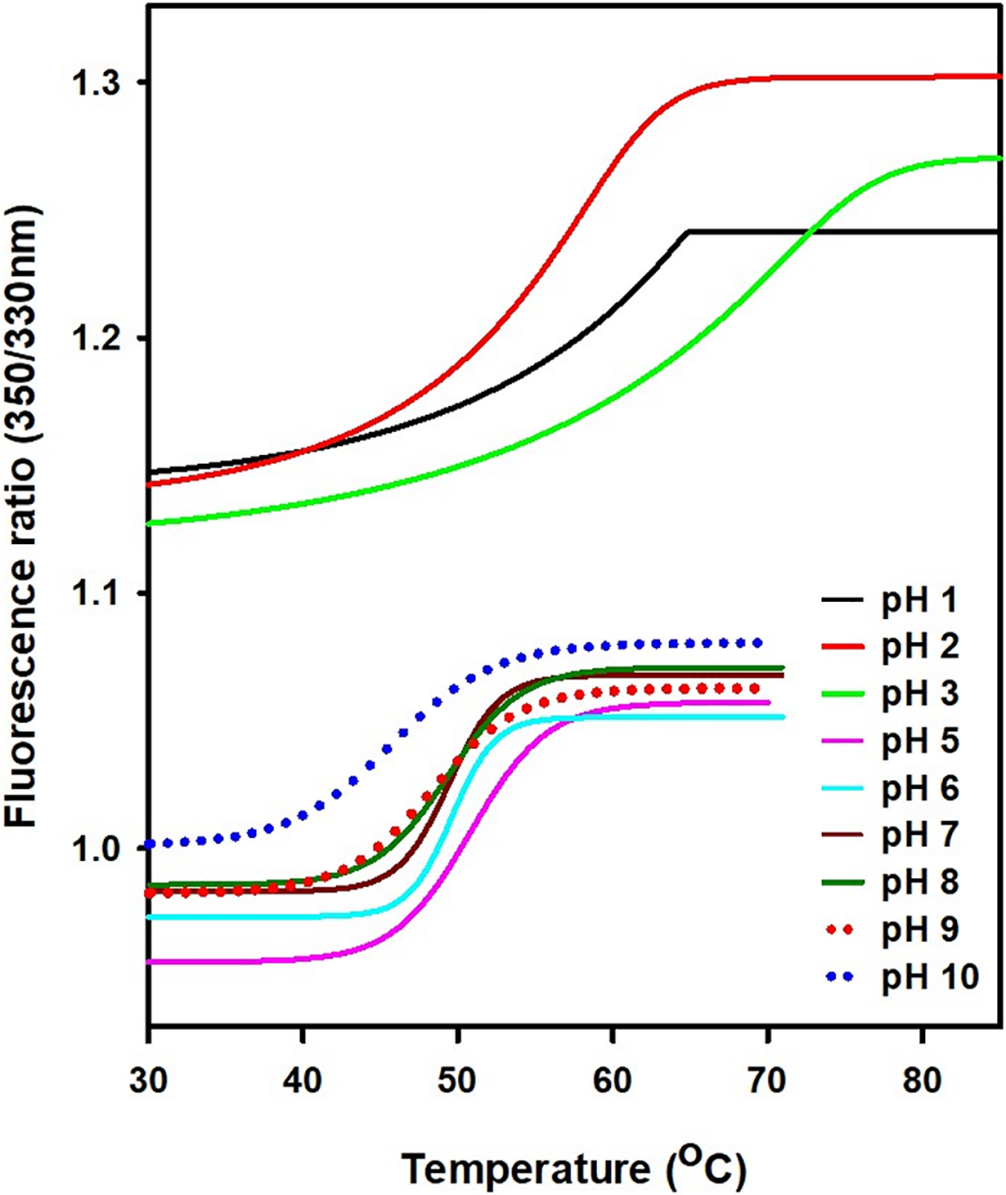
The thermal shift assay for *Cd*GSTM1 at pH 1.0–10.0. Intrinsic tryptophan fluorescence was used to determine the thermal stability of *Cd*GSTM1 at different pH values. *Cd*GSTM1 at pH 5.0 to 10.0 was continuously heated a rate of 1°C min^-1^ from 30 to 70°C, while *Cd*GSTM1 at pH 1.0 to 3.0 was heated from 30 to 85°C. Samples were excited at 295 nm (10 nm bandwidth) and emission at 330 and 350 nm (10 nm bandwidth) were recorded. The emission ratio of 350 nm to 330 nm was plotted with respect to temperature. *Cd*GSTM1 at different pH values undergoes thermal unfolding via a single transition. The mid-point of the thermal transition was identified as thermal melting point (*Tm*).

We have determined the thermal stability of *Cd*GSTM1 at pH 1.0–10.0 in response to changes in intrinsic tryptophan fluorescence. The temperature-melting curves of the samples were obtained through gradual heating at a constant rate of 1°C min^-1^. *Cd*GSTM1 formed visible aggregates above 75°C between pH 5.0 and 10.0. However, below pH 3.0, no aggregates were observed up to 94°C. Therefore, *Cd*GSTM1 samples between pH 5.0 and 10.0 were gradually heated till 70°C and samples below pH 3.0 were subjected to thermal denaturation till 94°C. The emission ratio of 350 nm to 330 nm was plotted with respect to temperature at the different pH values (Fig 10). The ratio of 350/330 nm was clustered in two groups owing to a shift in λ_max_ (6-nm red-shift) at pH ≤3.0.

*Cd*GSTM1 undergoes a single thermal transition at all pHs. The tertiary structure of *Cd*GSTM1 undergoes thermal denaturation via a two-state unfolding pathway at all pH values (Fig 10). In this study, thermal stability of *Cd*GSTM1 was moderate between pH 1.0 and 10.0 (Table 2). *Cd*GSTM1 was relatively more stable at an acidic pH than at neutral and alkaline pH. Exceptionally high stability (65.2°C) at pH 3.0 might have resulted from monomer-dimer equilibrium.

**Table 2:** Thermal melting point (*Tm*) of *Cd*GSTM1at pH 1.0–10.0.

pH	*Tm* (^o^C)
1.0	56.4
2.0	54.8
3.0	65.2
5.0	51.0
6.0	49.5
7.0	49.0
8.0	49.2
9.0	48.0
10.0	45.7

### Thermodynamic and spectroscopic properties of dimeric and monomeric *Cd*GSTM1 revealed through dynamic multimode spectroscopy

**Fig 11:**
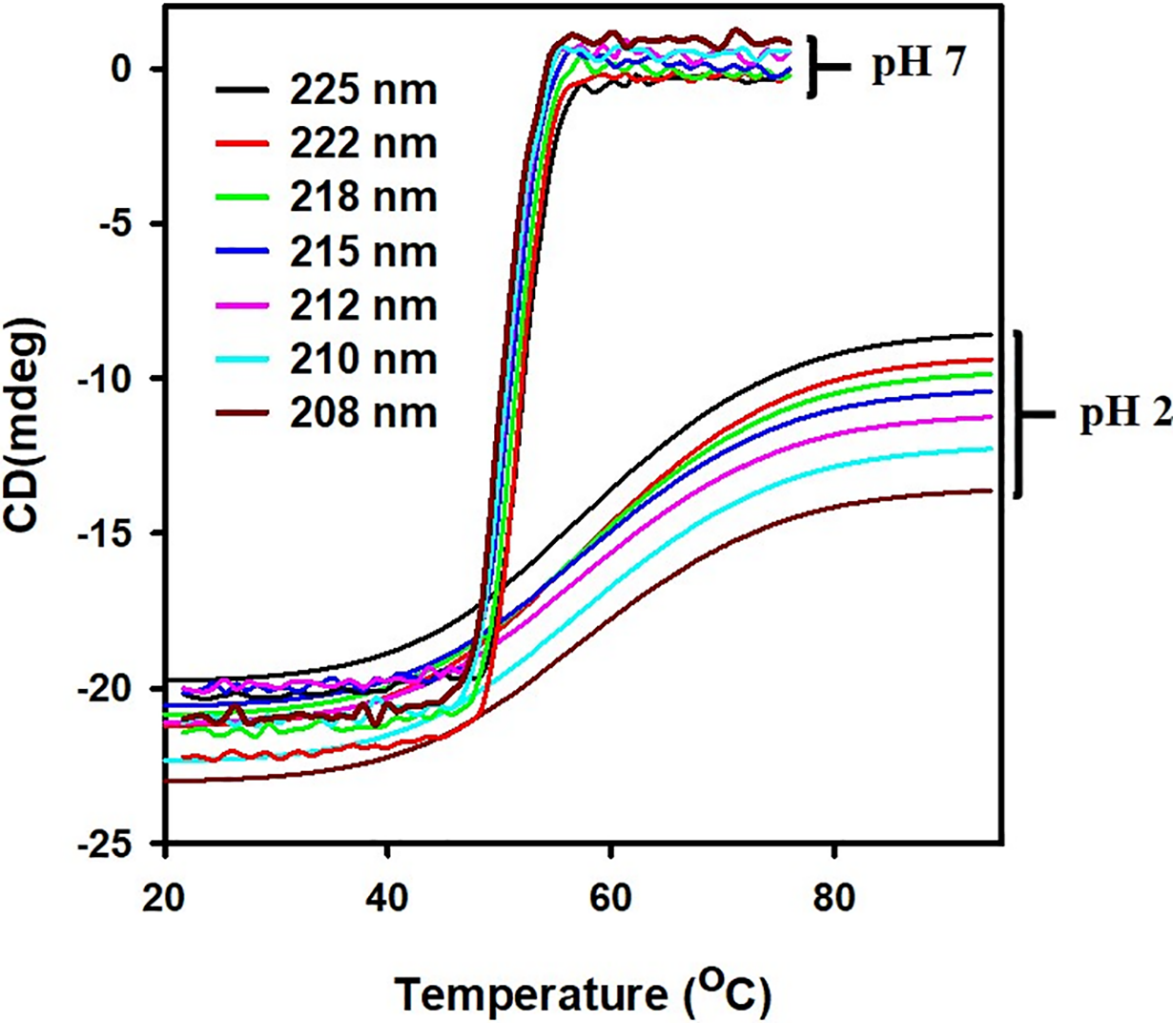
Temperature-dependent conformational changes in dimeric and monomeric *Cd*GSTM1 at different wavelengths. Far-UV circular dichroism spectra values represented in mdeg units and plotted as a function of temperature. Thermal transitions were processed using Global 3 software, especially developed to analyse dynamic multimode spectroscopy data. Wavelengths are colour-coded. Single thermal transitions are clearly visible at wavelengths between 208 and 225 nm.

Dynamic multimode spectroscopy is an information-rich experimental technique based on far-UV CD to determine changes in secondary structure in the entire temperature range and furnishes thermodynamic data [43, 44]. Therefore, the dimeric form (pH 7.0) and monomeric form (pH 2.0) of *Cd*GSTM1 were subjected to dynamic multimode spectroscopy (DMS) to evaluate structural and thermodynamic properties in both states. The samples at pH 7.0 were constantly heated (1°C min^-1^) from 20–75°C, while the sample at pH 2.0 was heated up to 94°C under identical conditions. Far-UV CD spectra of both samples were recorded between 200 and 260 nm as a function of temperature. Selected wavelengths are shown in Fig 11. Dimeric *Cd*GSTM1 (pH 7.0) and monomeric *Cd*HSTM1 (pH 2.0) unfold via a single transition. At pH 7.0, they undergo sharp thermal transitions and secondary structure is completely lost at 57°C, while *Cd*GSTM1 at pH 2.0 follows shallow thermal transition and retains nearly half of the secondary structure up to 94°C. The thermal melting points (*Tm*) and van't Hoff enthalpy of *Cd*GSTM1 at both pHs were determined via Global 3 analysis software, Applied Photophysics Ltd, UK. The *Tm* of dimeric *Cd*GSTM1 at pH 7.0 was 51.5 ± 0.1°C and van't Hoff enthalpy was 666.4 ± 9.8 kJ mol^-1^. The *Tm* of monomeric *Cd*GSTM1 was higher 54.2 ± 0.1°C; however, the van't Hoff enthalpy was quite low (116.5 ± 0.8 kJ mol^-1^). The thermal shift assay also yielded a single thermal transition with *Tm* values of 49.0 and 54.8°C at pH 7.0 and 2.0, respectively (Fig 10).

**Fig 12:**
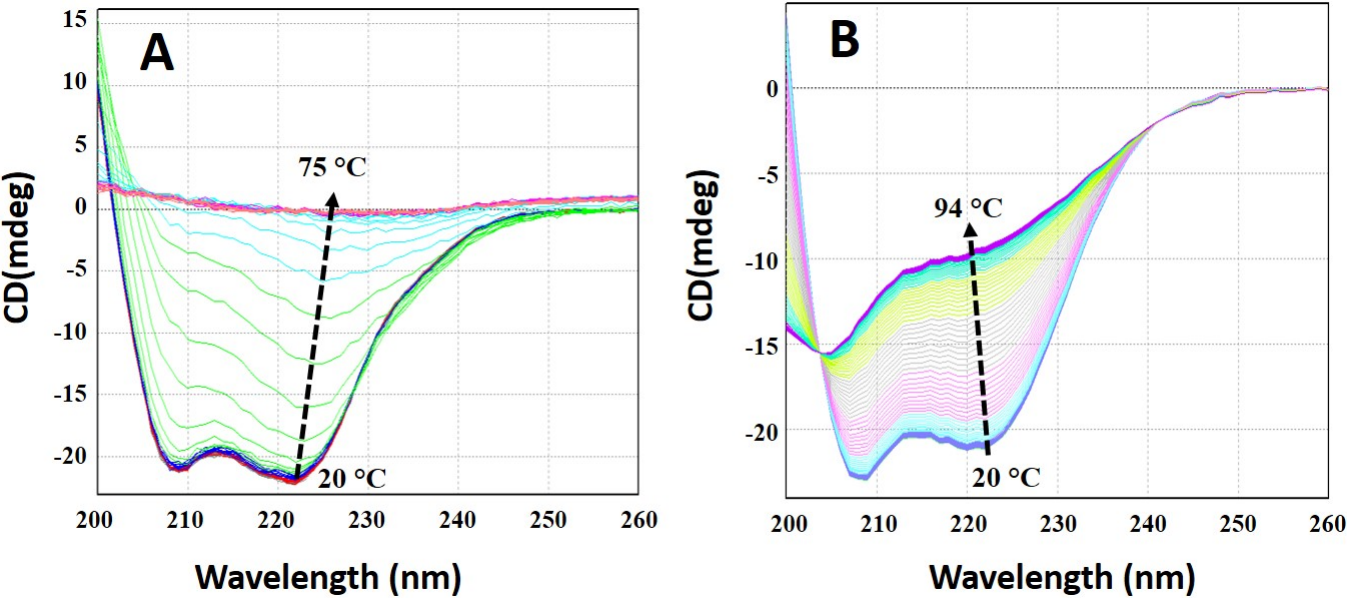
Far-UV circular dichroism (CD) spectra of *Cd*GSTM1 at different temperatures. **(A)** *Cd*GSTM1 (0.2 mg mL^-1^) at pH 7.0 was heat denatured at constant rate (1°C/min). Far-UV CD spectra were collected from 200–260 nm at intervals of 1°C from 20–75°C. **(B)** *Cd*GSTM1 (0.2 mg mL^-1^) at pH 2.0 was heat-denatured and data collected under identical condition from 20–94°C.

Changes in secondary structure conformation at different temperature are shown in Fig 12. At pH 7.0, loss of the secondary structure was observed beyond 45°C and was completely lost at 57°C. During thermal unfolding beyond 45°C, a 222-nm peak shifted towards single a 225-nm peak (Fig 12A). Thermal stress at pH 7.0 led to the conversion of *Cd*GSTM1 from an alpha helical structure into a cross-beta sheeted structure, which aggregated beyond 75°C [45]. However, thermal denaturation of monomeric *Cd*GSTM1 led to the conversion of the same alpha-helical structure to a random-coil structure (Fig 12B). Hence, monomeric *Cd*GSTM1 slowly loses its secondary structure beyond 30°C but retains nearly half of its secondary structure even up to 94°C.

**Fig 13:**
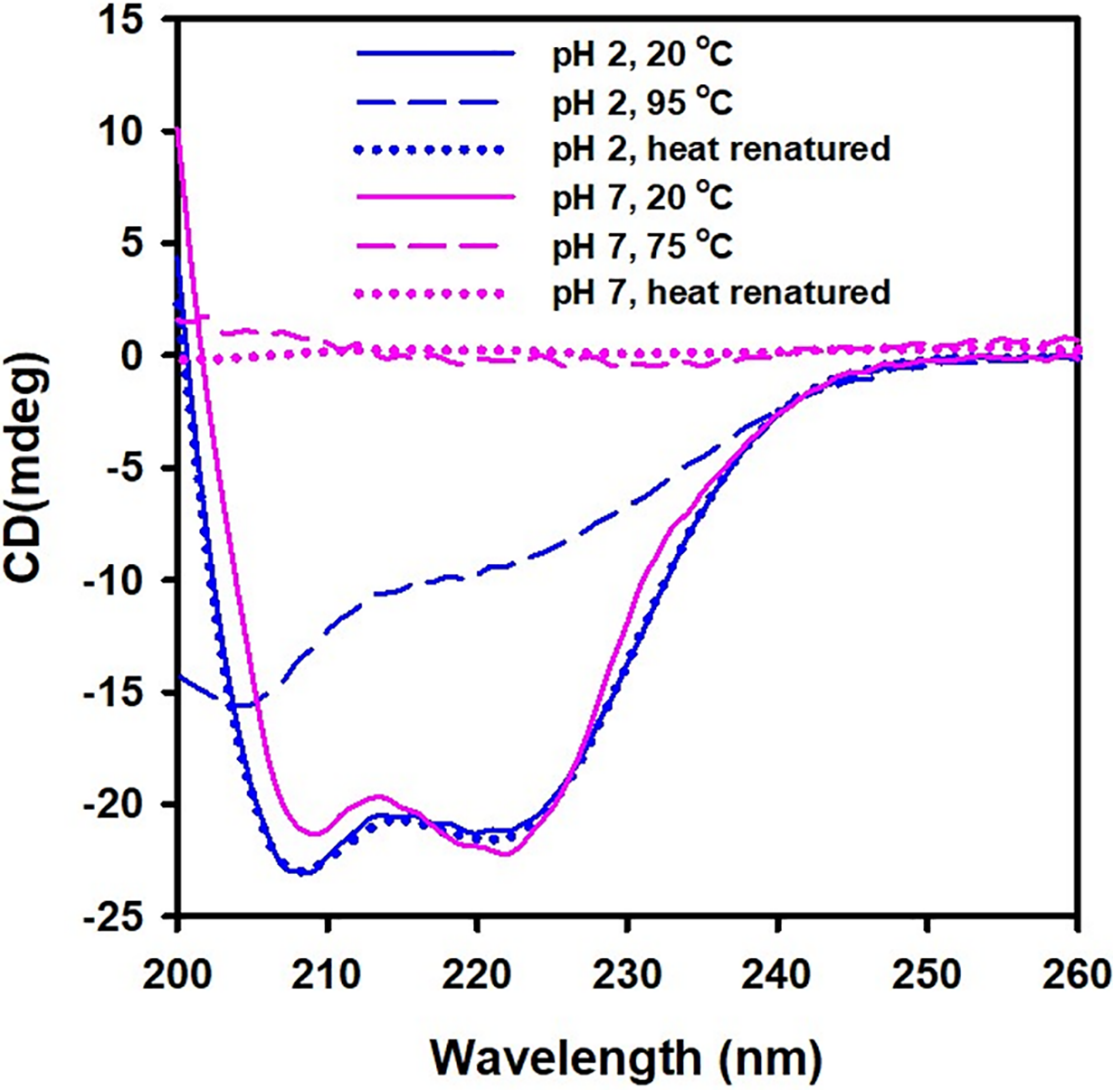
Reversibility of thermal unfolding of dimeric and monomeric *Cd*GSTM1. Far-UV CD of dimeric and monomeric *Cd*GSTM1 (0.2 mg mL^-1^) in the native state (20°C), denatured state (75 or 94°C), and renatured state (cooled to 20°C). At low temperature, *Cd*GSTM1 in the dimeric and monomeric states adopt an α-helix conformation. In the heat-stressed condition, *Cd*GSTM1 in the dimeric state loses its secondary structure completely, while the monomeric form retained nearly half of the secondary structure with a dominant random-coil conformation. The dimeric form of *Cd*GSTM1 was unable to refold, while the monomeric form refolded completely to its secondary structure.

Thereafter, we measured the reversibility of thermal unfolding in dimeric and monomeric *Cd*GSTM1. As shown in Fig 13, dimeric *Cd*GSTM1 at 20°C is an alpha-helical protein, which has completely lost its secondary structure at 75°C. Moreover, heat-denatured dimeric *Cd*GSTM1 could not refold. Monomeric *Cd*GSTM1 at pH 2.0 was also alpha-helical at 20°C, but converted into a completely reversible random-coiled dominant structure at 94°C. Far-UV CD spectra of heat-renatured monomeric *Cd*GSTM1 at pH 2.0 was superimposed on the native monomeric form of *Cd*GSTM1 at 20°C.

## Discussion

GSTs are structurally conserved and ubiquitous in virtually all organisms [22]. They are tremendously functionally divergent, which is an inevitable requisite for their role as major detoxification enzymes [46, 47]. The role of GSTs in multiple metabolic pathways and in stress responses in several organisms has been extensively studied [11, 48]. Moreover, GST has been used as a fusion tag for solubility enhancement, ease of purification, and other protein engineering purposes [49, 50]. Most GSTs are marginally thermostable at neutral pH. However, certain GSTs exhibit relatively higher thermo-stability and wider pH stability [51–53]. This study evaluated thermodynamic and conformational properties of the pH-induced structural changes in recombinant *Cd*GSTM1 via enzymatic assays, size-exclusion chromatography, intrinsic and extrinsic fluorescence, acrylamide quenching, far-UV CD, thermal shift assay, and dynamic multimode spectroscopy.

*Cd*GSTM1 was cloned in its native, soluble state in *E*. *coli*. Subsequently, it was purified to homogeneity, using affinity (GSH-agarose) and size-exclusion chromatography. The molecular weight of *Cd*GSTM1 was determined using SEC and was found to exist in the dimeric state in solution. Between pH 3.0 and 5.0 *Cd*GSTM1 undergoes a conformational change, resulting in nearly complete loss of enzyme activity. The crystal structure of Mu-class GST revealed that Tyr7 (catalytic residue) located at the NTD of one subunit interacts with the Phe57 located at the interface loop of the second subunit [20]. Destabilization of the interface loop, in turn, affects substrate binding and catalysis [54]. Hence, dimer interface interactions stabilize the loop conformation and are essential for enzyme activity.

Intrinsic tryptophan fluorescence provides information regarding polarity and hydrophobicity of the tryptophan microenvironment. It has been extensively used as a spectral probe to investigate protein tertiary structure [55]. Generally, interior tryptophan residues in folded proteins displayed fluorescence emission maximum below 340 nm, whereas upon protein unfolding, the fluorescence emission maximum red-shifted to 350–355 nm. *Cd*GSTM1 contains four tryptophan residues. A 3D model of *Cd*GSTM1 revealed that two of these (Trp8 and Trp46) are located proximal to the dimer interface (data not shown). The emission maximum of *Cd*GSTM1 at neutral pH was observed at 336 nm (Fig 4), suggesting that the tryptophan residues in the native conformation are significantly buried in the protein core. The tryptophan emission maxima were mostly unchanged from pH 5.0 to 10.0, suggesting that the tertiary structure of the protein was stable in this pH range (Fig 4). However, a sharp red-shift (6 nm) in the intrinsic emission maxima was observed between pH 3.0 and 5.0, with no further change in emission maxima below pH 3.0 (Fig 4). The red-shift in emission maxima resulted from changes in the tryptophan microenvironment from a non-polar to a polar environment during protein denaturation or dissociation of subunits of multimeric proteins or both [55]. A minor shift in emission maxima (from 336 to 342 nm) of *Cd*GSTM1 below pH 5.0 indicated a marginal change around the tryptophan residues in *Cd*GSTM1 at low pH. On treatment with a chemical denaturant (6 M GdnHCl), *Cd*GSTM1 undergoes complete unfolding with an emission maxima of 360 nm (data not shown).

Loss of enzyme activity seems to coincide with red-shift in emission maxima, indicating exposure of aromatic residues, especially tryptophan, to the polar environment. These data suggest the dissociation or denaturation, or both, of dimeric *Cd*GSTM1 below pH 5.0. An extrinsic fluorescence dye (ANS) does not fluoresce in an aqueous buffer but fluoresces when exposed to a hydrophobic environment. ANS does not bind or exhibit very low binding with the native and completely unfolded polypeptide chain; however, it binds with protein folding intermediates formed during unfolding [56]. ANS emission spectra, used to measure surface hydrophobicity, showed a blue-shift and a two-fold increase in fluorescence intensity between pH 3.0 and 5.0. Previously, ANS was found to bind the dimer interface, thereby decreasing the emission maximum from 535 nm to 490–500 nm, accompanied by an enhanced fluorescence signal [29, 30, 57]. The blue-shift in the ANS spectra revealed that the environment of the ANS-binding site is hydrophobic. RLS is routinely used to detect the quantity of aggregates in a solution [58, 59]. An increase in surface hydrophobicity did not affect the solubility of *Cd*GSTM1 at low pH.

The integrity of the *Cd*GSTM1 quaternary structure was assessed using SEC and glutaraldehyde cross-linking, which revealed that *Cd*GSTM1 dissociated into subunits at pH 4.0 and exists in a monomer-dimer equilibrium. However, at pH 2.0, it exists only as a monomer. Although 0.15 M NaCl was included in the gel filtration buffer to supress ionic interactions with the matrix, monomeric *Cd*GSTM1 had a retarding effect on the column. Therefore, we could not determine the molecular weight of the subunit.

The dimeric form often tends to contain Trp, Tyr, and Phe (large hydrophobic amino acid residues) at the dimer interface to anchor subunits [60]. A modelled structure of *Cd*GSTM1 revealed a conserved Phe57 residue at the dimer interface, which serves as the key of the hydrophobic lock-and-key motif (Fig 1B). The phenyl ring of Phe57 protrudes from the NTD of one subunit into a hydrophobic pocket generated within the C-terminal domain of the other subunit formed by alpha-helix 4 and 5 [36]. A mixed charge cluster (six positively and six negatively charged residues) in a 24-residue-long stretch (Arg78 to Glu101) located close to the dimer interface (data not shown) forms ionic interactions between subunits. Thus, dimeric *Cd*GSTM1 stabilized by charge-charge interaction and hydrophobic forces. The crystal structure of rat GSTM1-1 revealed that two groups of interactions (hydrophobic lock-and-key interaction motifs and ionic interactions) formed the core of the interface contacts and stabilized it [20, 26, 33, 54].

Far-UV CD spectroscopy has been extensively used in analysing the secondary structure of the protein under different conditions. In this study, the far-UV CD spectrum of *Cd*GSTM1 displayed a typical alpha-helix-structure dominant structure (Fig 5A). All the secondary structures (alpha helices, beta sheets, and beta-turns) contents were almost intact between pH 1.0 and 7.0. In the alkaline region, slight conversion of the alpha helix to the random-coil was observed (Fig 5B). Although *Cd*GSTM1 lost its activity at a low pH and changes in tertiary structure also occurred below pH 5.0, far-UV CD revealed that *Cd*GSTM1 retains nearly all structures below pH 5.0. These data suggest that *Cd*GSTM1 dissociates below pH 5.0 but does not denature. Monomeric *Cd*GSTM1 was folded and was soluble but inactive owing to the requirement of a substrate interface for substrate binding.

Thermal shift assay based on intrinsic tryptophan fluorescence and thermal transition based on far-UV CD spectra of the proteins are orthogonal techniques, which have been extensively used to monitor protein stability under different conditions [61, 62]. Thermal melting curves based on changes in the tertiary structure of *Cd*GSTM1 revealed that it follows two-state thermal unfolding at pH 1.0 to 10.0. The protein was moderately thermostable, and its thermostability increased significantly below pH 3.0. Thermal stability based on secondary structure (far-UV CD) also had similar *Tm* values and temperature-induced unfolding pathways of dimeric and monomeric *Cd*GSTM1 compared to those obtained with the thermal shift assay [62]. The present results show that dimeric *Cd*GSTM1 undergoes a sharp thermal transition with a large change in van't Hoff enthalpy (666.4 ± 9.8 kJ mol^-1^), while the monomeric form undergoes broad range of thermal transitions (approximately 40°C) with lower van't Hoff enthalpy 116.5 ± 0.8 kJ mol^-1^. Dimeric *Cd*GSTM1 attains cross-beta sheeted structures during thermal unfolding, which undergoes irreversible thermal aggregation. However, alpha helices in monomeric *Cd*GSTM1 were gradually converted to random-coil-like structures during thermal unfolding, which was completely reversible. Similar thermal unfolding behaviour was also observed in rat GSTM2-2, which unfolds via a single transition with a *Tm* of 54°C [36].

**Fig 14:**
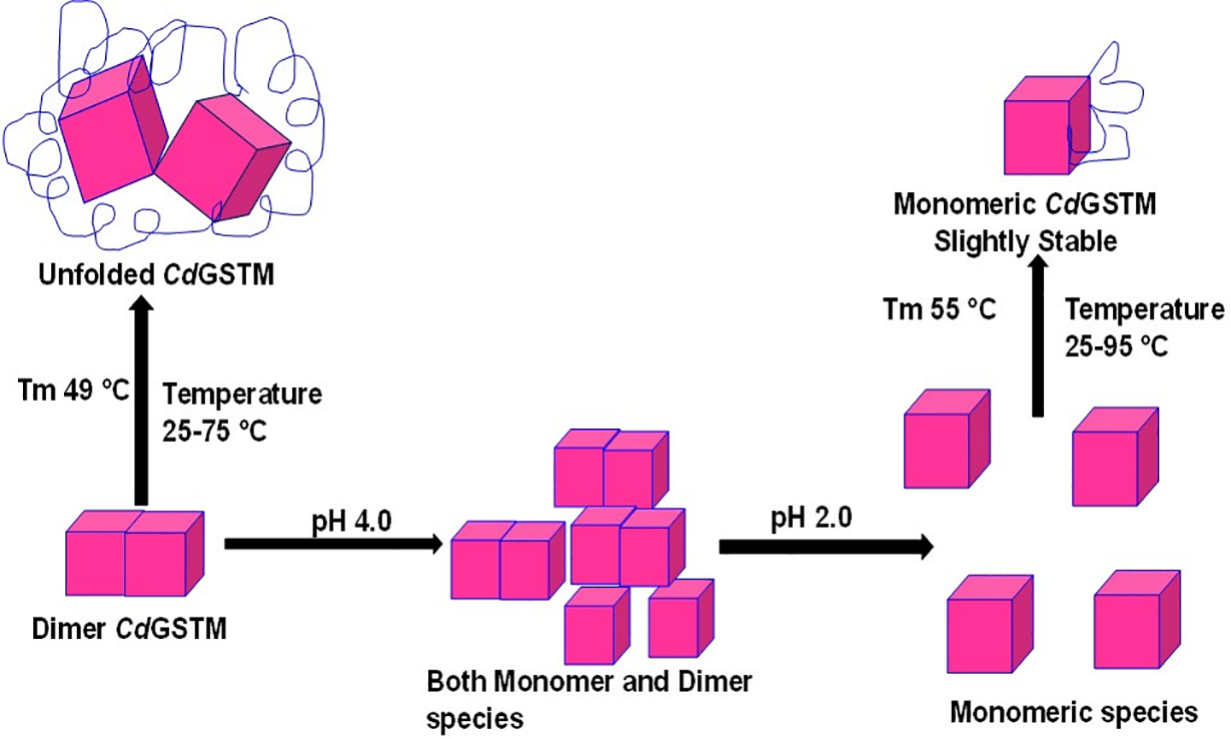
Graphical representation of pH-dependent monomerization, and conformational changes in *Cd*GSTM1 and temperature-induced unfolding of monomeric and dimeric *Cd*GSTM1.

Understanding protein stability is necessary to gain insights into protein folding, structure, and function. Proteins that are marginally stabilized exceed a minimal threshold to attain a native conformation and function [63, 64]. Generally, thermodynamic stabilities of most water-soluble globular proteins varies from 12 and 42 kJ/mol [64]. The protein is stabilized via a delicate balance between certain forces and interactions. Hydrophobic forces and electrostatic interactions are major factors determining protein stability. To balance optimum biological function and conformational stability, electrostatic interactions are optimised in proteins [65]. Analysis of pH-dependent protein stability helps understand electrostatic interactions in proteins and the contribution of specific charged residues in protein structure and function. The thermodynamic stability of proteins in the native and denatured state are linked to pKa groups of the side chains, which is pH-dependent. The pKa values of the residues in the native and unfolded states depend on several factors: charge-charge, H-bonds, charge-dipole, and desolvation effects. The degree of interactions between charged resides and the rest of the protein in the native or denatured forms determines the titration properties of the ionisable groups. Generally, proteins lose structure-function and stability below pH 5.0 and above pH 10.0 owing to ionization of buried ionisable residues (e.g., Tyr, His) in the folded native state. In the strong acidic or alkaline region, ionisable residues from the surface to the core of the proteins gradually get ionized and lead to protein unfolding. The relatively higher stability of monomeric *Cd*GSTM1 compared to that of dimeric *Cd*GSTM may have resulted from higher intra-chain interactions within the monomer. The overall data is schematically presented in Fig 14. Briefly, *Cd*GSTM1 exists in dimeric state at pH 7.0 while it equilibrated in dimer-monomer form at pH 4.0. At acidic pH of 2.0, it is populated in monomeric form. The dimeric form was having relatively less thermal stability than the monomeric form.

## Material and methods

### Materials

GSH-agarose was purchased from GenScript, USA. Superdex 75 and Superdex 200 increase-prepacked column was from GE Healthcare Life Sciences. All chemicals and kits were purchased from Sigma-Aldrich Chemical Company, St. Louis, MO, USA, Bio Basic Inc., Toronto, ON, Canada, New England Biolabs, MA, USA and Qiagen, CA, USA.

### Multiple sequence alignment and structural analysis

The homologous mammalian sequences of GSTM1 was identified using *Cd*GSTM1 (accession number XP_010974221.1) amino acid sequence in PSI-BLAST. Ten homologous sequences of *Cd*GSTM1 were retrieved from database. Multiple sequence alignment was done using MAFFT Multiple Sequence Alignment and Jalview [40, 66]. The output of aligned sequences was color-coded. Three–dimentional structure *Cd*GSTM1 was modelled using Swiss-model server [67] using human glutathione S-transferase *h*GSTM1a-1a (PDB: 1GTU) as a template. The 3D structure of GSTM1 was analyzed using PyMOL software [68].

### Expression of *Cd*GSTM1 in *Escherichia coli* BL21 (DE3)

Codons in the open reading frame (ORF) of *Cd*GSTM1 were optimized for expression in *E*. *coli* BL21(DE3), maintaining the same amino acid sequence encoded by the gene (accession number HM475154), cloned in pET-3a(+) expression vector under the control of T7 promotor (Genscript) and used to transform *E*. *coli* BL-21(DE3). Recombinant *Cd*GSTM1 was induced using in 1 L LB-modified medium supplemented with 2 g L^-1^ lactose, 5 g L^-1^ glycerol, 5 g L^-1^ yeast, 10 g L^-1^ peptone, 0.5 g L^-1^ glucose, 0.7 g L^-1^ sodium sulfate, 2.5 g L^-1^ ammonium chloride, 0.1 g L^-1^ magnesium chloride, 0.1 g L^-1^ calcium chloride, 0.1 g L^-1^ potassium chloride, and 100 μg mL^-1^ ampicillin. The inoculated medium was incubated at 30°C with continuous agitation (160 rpm). The biomass obtained from approximately 24 h of culturing was harvested via centrifugation (6,000 ×g, 15 min) and weighed (approximately 3.6 g L^-1^). Thereafter, it was re-suspended in 10 mL of lysis buffer; 50 mM potassium phosphate buffer (pH 8.0), containing 300 mM NaCl, 1 mM phenylmethylsulfonyl fluoride (PMSF), and 1 mM Dithiothreitol (DTT). Cells were lysed via sonication five times for 8 seconds each, in ice, using an ultrasonicator (Soniprep 150 plus, MSE, UK), followed by centrifugation at 16,000 ×g for 15 min, and the clear supernatant was collected.

### Purification of recombinant *Cd*GSTM1

Recombinant *Cd*GSTM1 was purified using GSH-agarose adsorbent (2 mL; Genscript). The matrix was firstly washed with 10 mL equilibration buffer (50 mM potassium phosphate buffer [pH 7.5], 300 mM NaCl, 1 mM PMSF, 1 mM DTT, and 5% glycerol). The crude homogenate (10 mL) was loaded onto the column, and unbound proteins were washed with 50 mM potassium phosphate buffer [pH 7.5] containing 300 mM NaCl. Elution of bound *Cd*GSTM1 was achieved with 50 mM Tris-HCl buffer [pH 8.0] and 10 mM of GSH.

Active fractions were pooled and subjected to size-exclusion chromatography to eliminate minor impurities and reduced glutathione. Superdex 75 16/600 GL prepacked column was equilibrated with 50 mM phosphate buffer containing 150 mM NaCl at pH 7.5. The active fraction of GSH-agarose elute was pooled and loaded on a column using a 5 mL superloop on an AKTA purification system. The purity of the gel filtration peak was analysed with a 4–15% mini-PROTEAN pre-casted TGX gel (Bio-rad) and stained with Coomassie Brilliant Blue G-250. The concentration of purified *Cd*GSTM1was estimated at spectrophotometrically at 280 nm, with a molar extinction coefficient of 39,880 M^−1^cm^−1^.

The molecular weight of the *Cd*GSTM1 was calculated using a Superdex 200 increase 10/300 GL Prepacked Tricorn Column, calibrated with five proteins: thyroglobulin (669 kDa), ferritin (440 kDa), aldolase (158 kDa), conalbumin (75 kDa), and ovalbumin (44 kDa). Purified *Cd*GSTM1 (2.5 mg mL^-1^) in 50 mM Phosphate buffer containing 150 mM NaCl at pH 7.0 was applied on the column using a 500 μL superloop. To determine the quaternary structure at different pH, 0.25 mg *Cd*GSTM1 at different pH (2.0, 4.0, and 7.0) were loaded on the column pre-equilibrated with buffer containing 150 mM NaCl at the aforementioned three values of pH.

### GST assay

*Cd*GSTM1 activity was determined spectrophotometrically based on the increase in UV absorbance at 340 nm at 25°C owing to the conjugation of GSH with 1-chloro-2,4 dinitrobenzene (CDNB) in accordance with a previously reported method [41], with an extinction coefficient of 9.6 mM^-1^ cm^-1^. The reaction was carried out in 1 mL of 0.1 M potassium phosphate buffer (pH 6.5), 1.0 mM GSH, and 1.0 mM CDNB dissolved in ethanol and the enzyme preparation. One unit of *Cd*GSTM1 activity was defined as the amount of GST that catalyses the formation of 1.0 μmole of thioether per min.

### Exposure of *Cd*GSTM1 to varying pH

*Cd*GSTM1 (0.2 mg mL^-1^) was equilibrated overnight with buffers pH 1.0 to 10.0 at 22°C. The following buffers (50 mM each) were used: KCl-HCl (pH 1.0); Gly-HCl (pH 2.0 and 3.0); acetate buffer (pH 4.0 and 5.0); phosphate buffer (pH 6.0 and 7.0); Tris-HCl (pH 8.0); Gly-HCl (pH 9.0 and 10).

### Fluorescence spectroscopy

Total and tryptophan fluorescence emission spectra were measured at 25°C in a Cary Eclipse Fluorescence Spectrophotometer (Agilent Technologies, California, USA), coupled with Peltier temperature controller. *Cd*GSTM1 (0.1 mg mL^-1^) at different pH (1.0–10.0) in a 10-mm path-length cuvette was excited at 295 nm (bandwidth, 5 nm each) for recording total and tryptophan fluorescence emission spectra, respectively. For aggregation studies of *Cd*GSTM1at different pH, the Rayleigh light scattering (RLS) method was used. *Cd*GSTM1 concentration at different pH was maintained at 0.1 mg mL^-1^. The *Cd*GSTM1 sample was excited at 500 nm, and emission was collected from 450–550 nm. The bandwidth of excitation and emission slit was 1.5 nm. Fluorescence intensity at 500 nm was plotted against different pH. The change in surface hydrophobicity with respect to pH was measured using 8-anilinonaphthalene-1-sulfonic acid (ANS) extrinsic fluorophore. Fifty-molar excess of ANS was added in the *Cd*GSTM1 samples at different pH and incubated at room temperature for 30 min. Subsequently, samples were excited at 380 nm (2.5 nm bandwidth), and emission of ANS-bound proteins was measured at 400–600 nm (5-nm bandwidth).

### Acrylamide quenching

Stepwise two μl of acrylamide quencher was added from the stock of 5.0 M into incubated protein (0.10 mg mL^-1^) samples. The quencher was added to the protein samples till saturation was achieved (0–0.12 M). The Trp residues of protein sample were excited at 295 nm, and the emission spectrum was recorded at 300–400 nm. Reduction in fluorescence intensity at wavelength maximum was analyzed using the Stern–Volmer equation.

$$\frac{Fo}{F}=1+Ksv[Q]$$ 1

where Fo and F are the fluorescence intensities at maximum wavelength in the absence and presence of the quencher, respectively, Ksv is the Stern–Volmer quenching constant, and [Q] is the concentration of the quencher.

### Thermal shift assay

Thermal stability of *Cd*GSTM1 at different pH was analysed by continuously heating samples at 1°C min^-1^ from 30–90°C. The actual temperature of the samples was monitored using an internal temperature probe. The samples were excited at 295 nm (10-nm bandwidth) and data were collected at 330, 340, and 350 nm (10-nm bandwidth).

### Circular dichroism spectroscopy

Far-UV CD measurements were obtained using a Chirascan Plus spectropolarimeter (Applied Photophysics Ltd, UK), coupled with a Peltier temperature controller. The instrument was calibrated with (1S)-(+)-10-camphorsulphonic acid. The far-UVCD spectra were measured at a *Cd*GSTM1 concentration of 0.2 mg mL^-1^ with a 1-mm path length cell at 25°C. Three spectra of each sample were obtained at 200–260 nm. Air and buffer blank were subtracted from each spectrum. Percent secondary structure content was calculated using CDNN software.

Dynamic multi-mode spectroscopy of *Cd*GSTM1 at pH 2.0 and 7.0 was performed with a Chirascan-Plus spectrophotometer. Protein samples (0.2 mg mL^-1^) equilibrated overnight at pH 2.0 and 7.0 were gradually heated at 1°C min^-1^, and far-UV CD spectra were recorded at 200–250 nm. The actual sample temperature was monitored using an internal temperature probe. Thermal melting curves were analysed using Chirascan’s Global 3 software, Applied Photophysics Ltd, UK.

### Glutaraldehyde cross-linking

Glutaraldehyde cross-linking was performed in accordance with a previously reported method [69] with slight modification. Briefly, 0.2 mL of *Cd*GSTM1 (0.5 mg mL^-1^) at pH 2.0, 4.0, and 7.0 were treated with 0.25% glutaraldehyde at room temperature for 15 min. Subsequently, cross-linking was quenched by addition of 0.2 M sodium borohydride and incubated for 20 min at room temperature. Thereafter, 0.005 mL of 10% sodium deoxycholate was added. The cross-linked protein was precipitated by decreasing the pH to 2.0–2.5 via addition of orthophosphoric acid. The precipitated protein was separated via centrifugation at 13,000 rpm for 10 min at 4°C. The pellet was dissolved in SDS-loading dye.

## References

[1] Oakley AJ. Glutathione transferases: new functions. Current opinion in structural biology. 2005;15(6):716–23. Epub 2005/11/03. 10.1016/j.sbi.2005.10.005 . 16263269

[2] Oakley A. Glutathione transferases: a structural perspective. Drug metabolism reviews. 2011;43(2):138–51. Epub 2011/03/25. 10.3109/03602532.2011.558093 . 21428697

[3] Dirr H, Reinemer P, Huber R. X-ray crystal structures of cytosolic glutathione S-transferases. Implications for protein architecture, substrate recognition and catalytic function. European journal of biochemistry. 1994;220(3):645–61. Epub 1994/03/15. . 8143720 10.1111/j.1432-1033.1994.tb18666.x

[4] Armstrong RN. Structure, catalytic mechanism, and evolution of the glutathione transferases. Chemical research in toxicology. 1997;10(1):2–18. Epub 1997/01/01. 10.1021/tx960072x . 9074797

[5] Mannervik B, Danielson UH. Glutathione transferases—structure and catalytic activity. CRC critical reviews in biochemistry. 1988;23(3):283–337. Epub 1988/01/01. . 3069329 10.3109/10409238809088226

[6] Salinas AE, Wong MG. Glutathione S-transferases—a review. Current medicinal chemistry. 1999;6(4):279–309. Epub 1999/04/02. . 10101214

[7] Deponte M. Glutathione catalysis and the reaction mechanisms of glutathione-dependent enzymes. Biochimica et biophysica acta. 2013;1830(5):3217–66. Epub 2012/10/06. 10.1016/j.bbagen.2012.09.018 . 23036594

[8] Cummins I, Dixon DP, Freitag-Pohl S, Skipsey M, Edwards R. Multiple roles for plant glutathione transferases in xenobiotic detoxification. Drug metabolism reviews. 2011;43(2):266–80. Epub 2011/03/24. 10.3109/03602532.2011.552910 . 21425939

[9] Armstrong RN. Glutathione S-transferases: reaction mechanism, structure, and function. Chemical research in toxicology. 1991;4(2):131–40. Epub 1991/03/01. . 1782341 10.1021/tx00020a001

[10] Laborde E. Glutathione transferases as mediators of signaling pathways involved in cell proliferation and cell death. Cell death and differentiation. 2010;17(9):1373–80. Epub 2010/07/03. 10.1038/cdd.2010.80 . 20596078

[11] Tew KD, Manevich Y, Grek C, Xiong Y, Uys J, Townsend DM. The role of glutathione S-transferase P in signaling pathways and S-glutathionylation in cancer. Free radical biology & medicine. 2011;51(2):299–313. Epub 2011/05/12. 10.1016/j.freeradbiomed.2011.04.013 ; PubMed Central PMCID: PMC3125017. 21558000 PMC3125017

[12] Hayes JD, Flanagan JU, Jowsey IR. Glutathione transferases. Annual review of pharmacology and toxicology. 2005;45:51–88. Epub 2005/04/12. 10.1146/annurev.pharmtox.45.120403.095857 . 15822171

[13] Jakobsson PJ, Morgenstern R, Mancini J, Ford-Hutchinson A, Persson B. Common structural features of MAPEG—a widespread superfamily of membrane associated proteins with highly divergent functions in eicosanoid and glutathione metabolism. Protein science: a publication of the Protein Society. 1999;8(3):689–92. Epub 1999/03/26. 10.1110/ps.8.3.689 ; PubMed Central PMCID: PMC2144274. 10091672 PMC2144274

[14] Bresell A, Weinander R, Lundqvist G, Raza H, Shimoji M, Sun TH, et al Bioinformatic and enzymatic characterization of the MAPEG superfamily. The FEBS journal. 2005;272(7):1688–703. Epub 2005/03/30. 10.1111/j.1742-4658.2005.04596.x . 15794756

[15] Oakley AJ, Harnnoi T, Udomsinprasert R, Jirajaroenrat K, Ketterman AJ, Wilce MC. The crystal structures of glutathione S-transferases isozymes 1–3 and 1–4 from Anopheles dirus species B. Protein science: a publication of the Protein Society. 2001;10(11):2176–85. Epub 2001/10/18. 10.1110/ps.ps.21201 ; PubMed Central PMCID: PMC2374065. 11604524 PMC2374065

[16] Hayes JD, Pulford DJ. The glutathione S-transferase supergene family: regulation of GST and the contribution of the isoenzymes to cancer chemoprotection and drug resistance. Critical reviews in biochemistry and molecular biology. 1995;30(6):445–600. Epub 1995/01/01. 10.3109/10409239509083491 . 8770536

[17] Sheehan D, Meade G, Foley VM, Dowd CA. Structure, function and evolution of glutathione transferases: implications for classification of non-mammalian members of an ancient enzyme superfamily. The Biochemical journal. 2001;360(Pt 1):1–16. Epub 2001/11/07. ; PubMed Central PMCID: PMC1222196. 11695986 10.1042/0264-6021:3600001 PMC1222196

[18] Nebert DW, Vasiliou V. Analysis of the glutathione S-transferase (GST) gene family. Human genomics. 2004;1(6):460–4. Epub 2004/12/21. 10.1186/1479-7364-1-6-460 ; PubMed Central PMCID: PMC3500200. 15607001 PMC3500200

[19] Kalita J, Shukla R, Shukla H, Gadhave K, Giri R, Tripathi T. Comprehensive analysis of the catalytic and structural properties of a mu-class glutathione s-transferase from Fasciola gigantica. Scientific reports. 2017;7(1):17547 Epub 2017/12/14. 10.1038/s41598-017-17678-3 ; PubMed Central PMCID: PMC5727538. 29235505 PMC5727538

[20] Ji X, Zhang P, Armstrong RN, Gilliland GL. The three-dimensional structure of a glutathione S-transferase from the mu gene class. Structural analysis of the binary complex of isoenzyme 3–3 and glutathione at 2.2-A resolution. Biochemistry. 1992;31(42):10169–84. Epub 1992/10/27. . 1420139 10.1021/bi00157a004

[21] Perbandt M, Burmeister C, Walter RD, Betzel C, Liebau E. Native and inhibited structure of a Mu class-related glutathione S-transferase from Plasmodium falciparum. The Journal of biological chemistry. 2004;279(2):1336–42. Epub 2003/09/16. 10.1074/jbc.M309663200 . 12972411

[22] Atkinson HJ, Babbitt PC. Glutathione transferases are structural and functional outliers in the thioredoxin fold. Biochemistry. 2009;48(46):11108–16. Epub 2009/10/22. 10.1021/bi901180v ; PubMed Central PMCID: PMC2778357. 19842715 PMC2778357

[23] Winayanuwattikun P, Ketterman AJ. Catalytic and structural contributions for glutathione-binding residues in a Delta class glutathione S-transferase. The Biochemical journal. 2004;382(Pt 2):751–7. Epub 2004/06/09. 10.1042/BJ20040697 ; PubMed Central PMCID: PMC1133834. 15182230 PMC1133834

[24] Sayed Y, Wallace LA, Dirr HW. The hydrophobic lock-and-key intersubunit motif of glutathione transferase A1-1: implications for catalysis, ligandin function and stability. FEBS letters. 2000;465(2–3):169–72. Epub 2000/01/13. . 10631328 10.1016/s0014-5793(99)01747-0

[25] Kaplan W, Husler P, Klump H, Erhardt J, Sluis-Cremer N, Dirr H. Conformational stability of pGEX-expressed Schistosoma japonicum glutathione S-transferase: a detoxification enzyme and fusion-protein affinity tag. Protein science: a publication of the Protein Society. 1997;6(2):399–406. Epub 1997/02/01. 10.1002/pro.5560060216 ; PubMed Central PMCID: PMC2143637. 9041642 PMC2143637

[26] Luo JK, Hornby JA, Wallace LA, Chen J, Armstrong RN, Dirr HW. Impact of domain interchange on conformational stability and equilibrium folding of chimeric class micro glutathione transferases. Protein science: a publication of the Protein Society. 2002;11(9):2208–17. Epub 2002/08/23. 10.1110/ps.0208002 ; PubMed Central PMCID: PMC2373595. 12192076 PMC2373595

[27] Hornby JA, Codreanu SG, Armstrong RN, Dirr HW. Molecular recognition at the dimer interface of a class mu glutathione transferase: role of a hydrophobic interaction motif in dimer stability and protein function. Biochemistry. 2002;41(48):14238–47. Epub 2002/11/27. . 12450388 10.1021/bi020548d

[28] Aceto A, Caccuri AM, Sacchetta P, Bucciarelli T, Dragani B, Rosato N, et al Dissociation and unfolding of Pi-class glutathione transferase. Evidence for a monomeric inactive intermediate. The Biochemical journal. 1992;285 (Pt 1):241–5. Epub 1992/07/01. ; PubMed Central PMCID: PMC1132772. 1637306 10.1042/bj2850241 PMC1132772

[29] Erhardt J, Dirr H. Native dimer stabilizes the subunit tertiary structure of porcine class pi glutathione S-transferase. European journal of biochemistry. 1995;230(2):614–20. Epub 1995/06/01. . 7607236

[30] Stevens JM, Hornby JA, Armstrong RN, Dirr HW. Class sigma glutathione transferase unfolds via a dimeric and a monomeric intermediate: impact of subunit interface on conformational stability in the superfamily. Biochemistry. 1998;37(44):15534–41. Epub 1998/11/04. 10.1021/bi981044b . 9799517

[31] Codreanu SG, Thompson LC, Hachey DL, Dirr HW, Armstrong RN. Influence of the dimer interface on glutathione transferase structure and dynamics revealed by amide H/D exchange mass spectrometry. Biochemistry. 2005;44(31):10605–12. Epub 2005/08/03. 10.1021/bi050836k . 16060669

[32] Wongsantichon J, Ketterman AJ. An intersubunit lock-and-key 'clasp' motif in the dimer interface of Delta class glutathione transferase. The Biochemical journal. 2006;394(Pt 1):135–44. Epub 2005/10/18. 10.1042/BJ20050915 ; PubMed Central PMCID: PMC1386011. 16225458 PMC1386011

[33] Zhu ZY, Karlin S. Clusters of charged residues in protein three-dimensional structures. Proceedings of the National Academy of Sciences of the United States of America. 1996;93(16):8350–5. Epub 1996/08/06. ; PubMed Central PMCID: PMC38674. 8710874 10.1073/pnas.93.16.8350 PMC38674

[34] Wallace LA, Sluis-Cremer N, Dirr HW. Equilibrium and kinetic unfolding properties of dimeric human glutathione transferase A1-1. Biochemistry. 1998;37(15):5320–8. Epub 1998/05/16. 10.1021/bi972936z . 9548764

[35] Dirr HW, Reinemer P. Equilibrium unfolding of class pi glutathione S-transferase. Biochemical and biophysical research communications. 1991;180(1):294–300. Epub 1991/10/15. . 1930226 10.1016/s0006-291x(05)81291-4

[36] Hornby JA, Luo JK, Stevens JM, Wallace LA, Kaplan W, Armstrong RN, et al Equilibrium folding of dimeric class mu glutathione transferases involves a stable monomeric intermediate. Biochemistry. 2000;39(40):12336–44. Epub 2000/10/04. . 11015213 10.1021/bi000176d

[37] Sacchetta P, Pennelli A, Bucciarelli T, Cornelio L, Amicarelli F, Miranda M, et al Multiple unfolded states of glutathione transferase bbGSTP1-1 by guanidinium chloride. Archives of biochemistry and biophysics. 1999;369(1):100–6. Epub 1999/08/27. 10.1006/abbi.1999.1324 . 10462444

[38] Wu H, Guang X, Al-Fageeh MB, Cao J, Pan S, Zhou H, et al Camelid genomes reveal evolution and adaptation to desert environments. Nature communications. 2014;5:5188 Epub 2014/10/22. 10.1038/ncomms6188 . 25333821

[39] Watson EE, Kochore HH, Dabasso BH. Camels and Climate Resilience: Adaptation in Northern Kenya. Human ecology: an interdisciplinary journal. 2016;44(6):701–13. Epub 2016/12/27. 10.1007/s10745-016-9858-1 ; PubMed Central PMCID: PMC5143358. 28018023 PMC5143358

[40] Katoh K, Standley DM. MAFFT multiple sequence alignment software version 7: improvements in performance and usability. Molecular biology and evolution. 2013;30(4):772–80. Epub 2013/01/19. 10.1093/molbev/mst010 ; PubMed Central PMCID: PMC3603318. 23329690 PMC3603318

[41] Habig WH, Pabst MJ, Jakoby WB. Glutathione S-transferases. The first enzymatic step in mercapturic acid formation. The Journal of biological chemistry. 1974;249(22):7130–9. Epub 1974/11/25. . 4436300

[42] Boscolo B, Leal SS, Salgueiro CA, Ghibaudi EM, Gomes CM. The prominent conformational plasticity of lactoperoxidase: a chemical and pH stability analysis. Biochimica et biophysica acta. 2009;1794(7):1041–8. Epub 2009/03/24. 10.1016/j.bbapap.2009.03.003 . 19303061

[43] Malik A, Fouad D, Labrou NE, Al-Senaidy AM, Ismael MA, Saeed HM, et al Structural and thermodynamic properties of kappa class glutathione transferase from Camelus dromedarius. International journal of biological macromolecules. 2016;88:313–9. Epub 2016/04/06. 10.1016/j.ijbiomac.2016.03.065 . 27044344

[44] Malik A, Haroon A, Jagirdar H, Alsenaidy AM, Elrobh M, Khan W, et al Spectroscopic and thermodynamic properties of recombinant heat shock protein A6 from Camelus dromedarius. European biophysics journal: EBJ. 2015;44(1–2):17–26. Epub 2014/11/15. 10.1007/s00249-014-0997-2 . 25395330

[45] Khan JM, Sharma P, Arora K, Kishor N, Kaila P, Guptasarma P. The Achilles' Heel of "Ultrastable" Hyperthermophile Proteins: Submillimolar Concentrations of SDS Stimulate Rapid Conformational Change, Aggregation, and Amyloid Formation in Proteins Carrying Overall Positive Charge. Biochemistry. 2016;55(28):3920–36. Epub 2016/06/23. 10.1021/acs.biochem.5b01343 . 27331826

[46] He G, Guan CN, Chen QX, Gou XJ, Liu W, Zeng QY, et al Genome-Wide Analysis of the Glutathione S-Transferase Gene Family in Capsella rubella: Identification, Expression, and Biochemical Functions. Frontiers in plant science. 2016;7:1325 Epub 2016/09/16. 10.3389/fpls.2016.01325 ; PubMed Central PMCID: PMC5005422. 27630652 PMC5005422

[47] Liu YJ, Han XM, Ren LL, Yang HL, Zeng QY. Functional divergence of the glutathione S-transferase supergene family in Physcomitrella patens reveals complex patterns of large gene family evolution in land plants. Plant physiology. 2013;161(2):773–86. Epub 2012/11/29. 10.1104/pp.112.205815 ; PubMed Central PMCID: PMC3561018. 23188805 PMC3561018

[48] Singh S. Cytoprotective and regulatory functions of glutathione S-transferases in cancer cell proliferation and cell death. Cancer chemotherapy and pharmacology. 2015;75(1):1–15. Epub 2014/08/22. 10.1007/s00280-014-2566-x . 25143300

[49] Harper S, Speicher DW. Expression and purification of GST fusion proteins. Current protocols in protein science. 2008;Chapter 6:Unit 6 Epub 2008/05/21. 10.1002/0471140864.ps0606s52 . 18429193

[50] Smith DB, Johnson KS. Single-step purification of polypeptides expressed in Escherichia coli as fusions with glutathione S-transferase. Gene. 1988;67(1):31–40. Epub 1988/07/15. . 3047011 10.1016/0378-1119(88)90005-4

[51] Johansson AS, Stenberg G, Widersten M, Mannervik B. Structure-activity relationships and thermal stability of human glutathione transferase P1-1 governed by the H-site residue 105. Journal of molecular biology. 1998;278(3):687–98. Epub 1998/06/20. 10.1006/jmbi.1998.1708 . 9600848

[52] Dragani B, Cocco R, Principe DR, Cicconetti M, Aceto A. Structural characterization of acid-induced intermediates of human glutathione transferase P1-1. The international journal of biochemistry & cell biology. 2000;32(7):725–36. Epub 2000/06/17. . 10856703 10.1016/s1357-2725(00)00018-2

[53] Allocati N, Masulli M, Pietracupa M, Federici L, Di Ilio C. Evolutionarily conserved structural motifs in bacterial GST (glutathione S-transferase) are involved in protein folding and stability. The Biochemical journal. 2006;394(Pt 1):11–7. Epub 2005/10/27. 10.1042/BJ20051367 ; PubMed Central PMCID: PMC1385997. 16248855 PMC1385997

[54] Thompson LC, Walters J, Burke J, Parsons JF, Armstrong RN, Dirr HW. Double mutation at the subunit interface of glutathione transferase rGSTM1-1 results in a stable, folded monomer. Biochemistry. 2006;45(7):2267–73. Epub 2006/02/16. 10.1021/bi0519506 . 16475815

[55] Lackowicz JR. Principles of fluorescence spectroscopy. New York 2004;Plenum Press;. 10.1038/cdd.2010.80 PubMed PMID: 20596078.

[56] Engelhard M, Evans PA. Kinetics of interaction of partially folded proteins with a hydrophobic dye: evidence that molten globule character is maximal in early folding intermediates. Protein science: a publication of the Protein Society. 1995;4(8):1553–62. Epub 1995/08/01. 10.1002/pro.5560040813 ; PubMed Central PMCID: PMC2143185. 8520481 PMC2143185

[57] Sluis-Cremer N, Naidoo NN, Kaplan WH, Manoharan TH, Fahl WE, Dirr HW. Determination of a binding site for a non-substrate ligand in mammalian cytosolic glutathione S-transferases by means of fluorescence-resonance energy transfer. European journal of biochemistry. 1996;241(2):484–8. Epub 1996/10/15. . 8917446 10.1111/j.1432-1033.1996.00484.x

[58] Khan JM, Abdulrehman SA, Zaidi FK, Gourinath S, Khan RH. Hydrophobicity alone can not trigger aggregation in protonated mammalian serum albumins. Physical chemistry chemical physics: PCCP. 2014;16(11):5150–61. Epub 2014/02/01. 10.1039/c3cp54941k . 24481490

[59] Vetri V, Militello V. Thermal induced conformational changes involved in the aggregation pathways of beta-lactoglobulin. Biophysical chemistry. 2005;113(1):83–91. Epub 2004/12/25. 10.1016/j.bpc.2004.07.042 . 15617813

[60] Jones S, Thornton JM. Principles of protein-protein interactions. Proceedings of the National Academy of Sciences of the United States of America. 1996;93(1):13–20. Epub 1996/01/09. ; PubMed Central PMCID: PMC40170. 8552589 10.1073/pnas.93.1.13 PMC40170

[61] Malik A, Alsenaidy MA. MERS-CoV papain-like protease (PL(pro)): expression, purification, and spectroscopic/thermodynamic characterization. 3 Biotech. 2017;7(2):100 Epub 2017/06/01. 10.1007/s13205-017-0744-3 ; PubMed Central PMCID: PMC5449288. 28560640 PMC5449288

[62] Malik A, Albogami S, Alsenaidy AM, Aldbass AM, Alsenaidy MA, Khan ST. Spectral and thermal properties of novel eye lens zeta-crystallin. International journal of biological macromolecules. 2017;102:1052–8. Epub 2017/05/04. 10.1016/j.ijbiomac.2017.04.101 . 28465175

[63] Bloom JD, Raval A, Wilke CO. Thermodynamics of neutral protein evolution. Genetics. 2007;175(1):255–66. Epub 2006/11/18. 10.1534/genetics.106.061754 ; PubMed Central PMCID: PMC1775007. 17110496 PMC1775007

[64] DePristo MA, Weinreich DM, Hartl DL. Missense meanderings in sequence space: a biophysical view of protein evolution. Nature reviews Genetics. 2005;6(9):678–87. Epub 2005/08/03. 10.1038/nrg1672 . 16074985

[65] Foit L, Morgan GJ, Kern MJ, Steimer LR, von Hacht AA, Titchmarsh J, et al Optimizing protein stability in vivo. Molecular cell. 2009;36(5):861–71. Epub 2009/12/17. 10.1016/j.molcel.2009.11.022 ; PubMed Central PMCID: PMC2818778. 20005848 PMC2818778

[66] Waterhouse AM, Procter JB, Martin DM, Clamp M, Barton GJ. Jalview Version 2—a multiple sequence alignment editor and analysis workbench. Bioinformatics. 2009;25(9):1189–91. Epub 2009/01/20. 10.1093/bioinformatics/btp033 ; PubMed Central PMCID: PMC2672624. 19151095 PMC2672624

[67] Waterhouse A, Bertoni M, Bienert S, Studer G, Tauriello G, Gumienny R, et al SWISS-MODEL: homology modelling of protein structures and complexes. Nucleic acids research. 2018;46(W1):W296–W303. Epub 2018/05/23. 10.1093/nar/gky427 ; PubMed Central PMCID: PMC6030848. 29788355 PMC6030848

[68] The PyMOL Molecular Graphics System VS, LLC.

[69] Liang Y, Du F, Sanglier S, Zhou BR, Xia Y, Van Dorsselaer A, et al Unfolding of rabbit muscle creatine kinase induced by acid. A study using electrospray ionization mass spectrometry, isothermal titration calorimetry, and fluorescence spectroscopy. The Journal of biological chemistry. 2003;278(32):30098–105. Epub 2003/05/29. 10.1074/jbc.M304050200 . 12771138

